# Text messaging for maternal and infant retention in prevention of mother-to-child HIV transmission services: A pragmatic stepped-wedge cluster-randomized trial in Kenya

**DOI:** 10.1371/journal.pmed.1002924

**Published:** 2019-10-02

**Authors:** Thomas A. Odeny, James P. Hughes, Elizabeth A. Bukusi, Eliud Akama, Elvin H. Geng, King K. Holmes, R. Scott McClelland

**Affiliations:** 1 Department of Medicine, University of Missouri-Kansas City, Kansas City, Missouri, United States of America; 2 Center for Microbiology Research, Kenya Medical Research Institute, Nairobi, Kenya; 3 Department of Epidemiology, University of Washington, Seattle, Washington, United States of America; 4 Department of Biostatistics, University of Washington, Seattle, Washington, United States of America; 5 Department of Global Health, University of Washington, Seattle, Washington, United States of America; 6 Department of Obstetrics and Gynecology, University of Washington, Seattle, Washington, United States of America; 7 University of California San Francisco, San Francisco, California, United States of America; 8 Department of Medicine, University of Washington, Seattle, Washington, United States of America; 9 Center for AIDS Research, University of Washington, Seattle, Washington, United States of America; 10 Department of Microbiology, University of Washington, Seattle, Washington, United States of America; 11 Institute of Tropical and Infectious Diseases, University of Nairobi, Nairobi, Kenya; University of Southampton, UNITED KINGDOM

## Abstract

**Background:**

Timely diagnosis of infant HIV infection is essential for antiretroviral therapy (ART) initiation. In a randomized controlled trial, we found the Texting Improves Testing (TextIT) intervention (a theory-based text messaging system) to be efficacious for improving infant HIV testing rates and maternal retention in prevention of mother-to-child HIV transmission (PMTCT) programs. Using an implementation science approach, we aimed to evaluate real-world effectiveness of the intervention.

**Methods and findings:**

In a pragmatic, cluster-randomized, stepped-wedge trial with 2 time periods of observation, we randomly allocated 10 clinics to begin implementing the intervention immediately and 10 clinics to begin implementing 6 months later. To approximate real-world conditions, inclusion criteria were broad. Women at clinics implementing the intervention received up to 14 text messages during pregnancy and after delivery and had the option to respond to text messages, call, or send inquiry text messages to a designated clinic phone. The primary outcomes were infant HIV testing and maternal retention in care during the first 8 weeks after delivery. We used modified Poisson regression with robust variance estimation to estimate the relative risk and 95% confidence intervals (CIs). Generalized estimating equations were applied on individual-level data to account for clustering by site. Between February 2015 and December 2016, 4,681 women were assessed for study participation, and 2,515 were included. Participant characteristics at enrollment did not differ by study arm. Overall median age was 27 years (interquartile range [IQR] 23–30), median gestational age was 30 weeks (IQR 28–34), 99% were receiving ART, and 87% who enrolled during intervention phases owned a phone. Of 2,326 infants analyzed, 1,466 of 1,613 (90.9%) in the intervention group and 609 of 713 (85.4%) in the control group met the primary outcome of HIV virologic testing performed before 8 weeks after birth (adjusted relative risk [aRR] 1.03; 95% CI 0.97–1.10; *P* = 0.3). Of 2,472 women analyzed, 1,548 of 1,725 (90%) in the intervention group and 571 of 747 (76%) in the control group met the primary outcome of retention in care during the first 8 weeks after delivery (aRR 1.12; 95% CI 0.97–1.30; *P* = 0.1). This study had two main limitations. Staff at all facilities were aware of ongoing observation, which may have contributed to increased rates of infant HIV testing and maternal retention in care at both intervention and control facilities, and programmatic initiatives to improve maternal and infant retention in care were ongoing at all facilities at the time of this study, which likely limited the ability to demonstrate effectiveness of the trial intervention.

**Conclusions:**

In this study, a larger proportion of infants in the intervention group received HIV testing compared with the control group, but the difference was small and not statistically significant. There was also a nonsignificant increase in maternal postpartum retention in the intervention periods. Despite the lack of a significant effect of the intervention, key lessons emerged, both for strengthening PMTCT and for implementation research in general. Perhaps most important, improving the implementation of usual care may have been sufficient to substantially improve infant HIV testing rates.

**Trial registration:**

ClinicalTrials.gov Trial Number NCT02350140.

## Abstract 2

In a study in Kenya, Thomas Odeny and co-workers investigate the value of text reminders for infant HIV testing and retaining mothers in care.

## Author summary

**Why was this study done?:**

- Low retention of mother–infant pairs in postpartum HIV care poses a major threat to successful elimination of mother-to-child HIV transmission in sub-Saharan Africa.
- The increase in the number of mobile phone connections in sub-Saharan Africa has been spectacular, presenting a unique opportunity to add mobile technology for health (mHealth) to the armamentarium for turning the tide of HIV.
- We developed the Texting Improves Testing (TextIT) intervention to improve uptake of infant HIV testing and maternal retention in postpartum HIV care in a high–HIV-burden setting. Text messages were designed using behavioral theory. Women received up to 14 text messages during pregnancy and after delivery. Messages were tailored based on recipient’s gestational age, name, preferred time, desired language (English, Kiswahili, or Dholuo), date of delivery, and infant’s name. Participants had the option to respond to text messages, call, or send inquiry text messages.
- In a randomized controlled efficacy trial, we found that the TextIT intervention significantly improved rates of infant HIV testing and maternal retention in postpartum HIV care.
- To achieve policy change leading to broader implementation of this evidence-based intervention, we sought to provide evidence of effectiveness under real-world routine care conditions.

**What did the researchers do and find?:**

- Using an implementation science approach, we designed a cluster randomized, stepped-wedge trial with 2 time periods of observation. We randomly allocated 10 health facilities to begin implementing the intervention immediately, while the remaining 10 began implementing approximately 6 months later.
- Consistent with real-world implementation conditions, inclusion criteria were minimal. Women were offered the opportunity to receive text messages if they were ≥18 years old or emancipated minors and between 28 weeks’ gestation and delivery.
- We found that 1,466 of 1,613 (90.9%) infants in the intervention group underwent HIV testing within 8 weeks after birth compared to 609 of 713 (85.4%) in the control group.
- We also found that 1,548 of 1,725 (90%) women in the intervention group were retained in care during the first 8 weeks after delivery compared to 571 of 747 (76%) in the control group.

**What do these findings mean?:**

- We observed nonsignificant increases in infant HIV testing and maternal retention in postpartum care associated with short message service (SMS) text messaging. Nevertheless, HIV testing of >90% aligns with the global target of diagnosing >90% of all children living with HIV.
- Recognizing that strict adherence to statistical thresholds in implementation research may not provide optimal guidance for public health practice, interpretation of estimates of improvement in such studies should be contextualized in terms of the costs and benefits of implementing at scale.

## Introduction

Timely antiretroviral therapy (ART) initiation among children reduces mortality and limits morbidity associated with HIV [1]. Timely diagnosis of HIV infection among infants is essential for successful ART initiation, yet only about 51% of HIV-exposed infants (HEIs) in the highest-burden countries were tested by 2 months of age in 2016 [2]. Moreover, approximately 50% of mother–infant pairs are lost to follow-up during the postpartum period [3].

We designed an interactive two-way theory-based text messaging intervention (referred to as the TextIT intervention, derived from **Text**ing **I**mproves **T**esting) to increase uptake of infant HIV testing by leveraging the rapid rise in mobile phone connections in Kenya [4]. The TextIT intervention included up to 14 text messages sent during pregnancy and after delivery. Messages were tailored based on recipient’s gestational age, name, preferred time, desired language (English, Kiswahili, or Dholuo), date of delivery, and infant’s name. Participants had the option to respond to text messages, call, or send inquiry text messages to a designated clinic phone. In a randomized controlled trial, we found the TextIT intervention to be efficacious for improving both infant HIV testing rates and maternal postpartum retention in prevention of mother-to-child transmission of HIV (PMTCT) programs [5]. Achieving policy change that would lead to broader implementation of this intervention would require evidence of effectiveness under real-world routine care conditions. To address this gap, we investigated the real-world applicability of TextIT using an implementation science approach [6]. We aimed to evaluate real-world effectiveness of the intervention for increasing rates of early infant diagnosis (EID) of HIV and increasing the proportion of HIV-infected pregnant women who remain in care outside of a controlled research setting.

## Methods

### Study design

This study was a pragmatic, cluster randomized, stepped-wedge trial with 2 observation time periods (S1 Trial protocol and S1 CONSORT checklist). Twenty clusters were selected for implementation. Ten clusters were randomly allocated to begin implementing the intervention immediately, while the remaining 10 began implementing approximately 6 months later.

### Participants

Study clusters were defined as public health facilities supported by the Family AIDS Care and Educational Services (FACES) program to provide PMTCT services in Kenya. FACES is a United States President’s Emergency Plan for AIDS Relief (PEPFAR)/Centers for Disease Control and Prevention (CDC) funded HIV prevention, care, and treatment program operated jointly by the Kenya Medical Research Institute and the University of California, San Francisco. At the time of this trial, FACES supported 136 government health facilities, spread across 3 counties (Kisumu, Migori, and Homa Bay) in the Nyanza region of Kenya. This region had the highest HIV prevalence in the country (15%) [7]. The top 20 clusters by patient volume (number of newly infected HIV-positive pregnant women in the prior 6 months) were selected for study inclusion. Potential participants included all HIV-positive pregnant women enrolled in the PMTCT program at target health facilities. To approximate real-world conditions, inclusion criteria were broad, with the intent of minimizing exclusion of subpopulations such as those without mobile phones. Women were offered the opportunity to receive text messages if they were ≥18 years old or emancipated minors and between 28 weeks’ gestation and delivery. Similar criteria were used to abstract data for women at control facilities. Women in the intervention group who reported sharing phones but had not disclosed their HIV status to the person sharing the phone did not receive the short message service (SMS) intervention but were included in the intention-to-treat analysis of outcomes.

### Randomization

**Fig 1:**
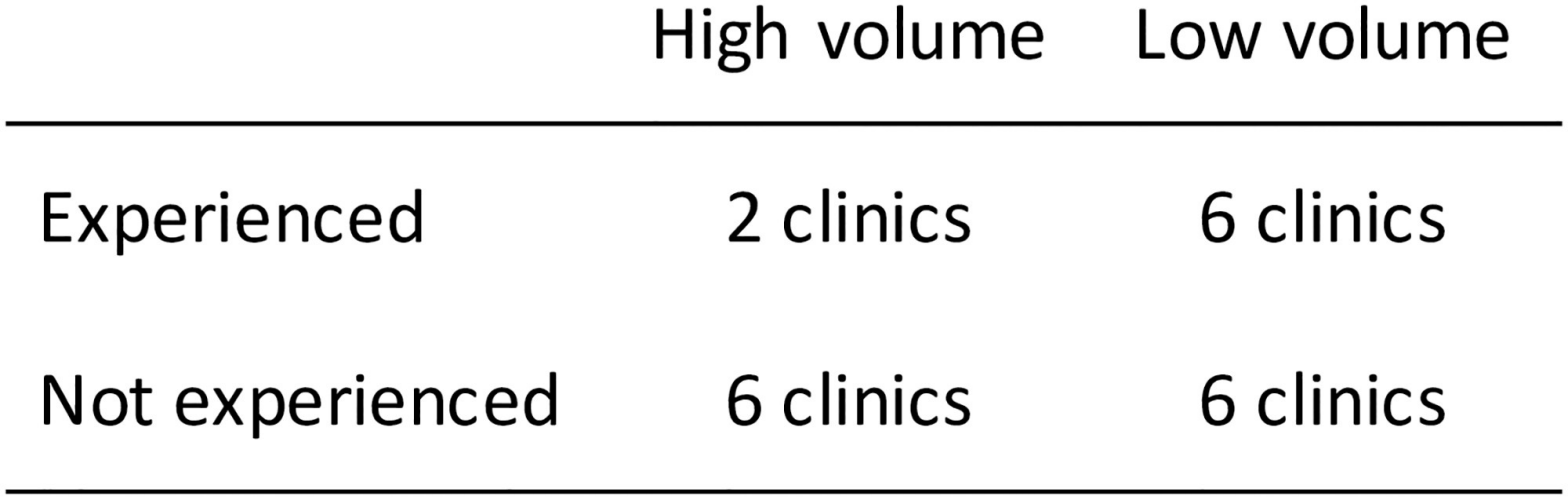
Stratification of study clusters based on volume and prior experience with implementing the intervention. Clinic volume was measured by the average number of pregnant women newly enrolled into the PMTCT program in the 12 months prior to randomization (≥50 versus <50 enrollments per month). Facilities were considered to have prior experience with TextIT if they had been part of a 6-month pilot project run immediately after release of the initial trial’s results. The 4 strata included (1) experienced and high volume, (2) experienced and low volume, (3) not experienced and high volume, and (4) not experienced and low volume. Within each stratum, half of clinics were randomly assigned to begin implementing the intervention immediately, while the other half began implementing approximately 6 months later. PMTCT, prevention of mother-to-child HIV transmission.

An independent biostatistician at the University of Washington’s Center for AIDS Research generated the randomization sequence and assigned clusters to intervention start periods. Randomization was stratified by clinic volume and experience level as shown in Fig 1. Clinic volume was measured by the average number of pregnant women newly enrolled into the PMTCT program in the 12 months prior to randomization (≥50 versus <50 enrollments per month). Facilities were considered to have prior experience with TextIT if they had been part of a 6-month pilot project run by FACES immediately after release of the initial trial’s results [5]. Facilities with prior experience did not continue to implement TextIT after the 6-month pilot project and were not implementing it at the time of randomization. Within each stratum, an equal number of clinics were randomly assigned to immediate or delayed implementation of the intervention. Due to the nature of the intervention and the need to inform facilities of their participation, it was not possible to blind clusters, healthcare providers, investigators, data analysts, or individual participants to group assignments.

### Training of implementers at intervention health facilities

“Mentor mothers” at each health facility abstracted data from routine clinic records and registered women to receive text messages during intervention periods. Mentor mothers are a formal lay cadre of women living with HIV who have recent experience in PMTCT (during the past 6 months to 2 years.) They are deployed to maternal and child health (MCH) clinics to share best practices of PMTCT with their peers attending care as part of the Ministry of Health–approved Kenya Mentor Mother Program [8]. Mentor mothers at phase I intervention sites were trained immediately after randomization, while those at phase II intervention sites were trained at the end of their control period, immediately prior to implementing the intervention. Training was provided in 2 stages. The first stage was a 2-day, conference-based training at a central location, attended only by mentor mothers. The second stage was a 1-day, facility-based training for all MCH service providers at each health facility. The 2-day training for mentor mothers included a review of PMTCT services and national guidelines, overview of the study protocol, training on research ethics, data collection using a mobile phone-based system, and procedures for registering patients to receive SMS text messages from the automated text messaging software. At the end of the 2-day training, an evaluation of competence was conducted. Each mentor mother was required to obtain certification of research ethics training by taking an online course following the 2-day training. The second stage of training was completed on site at each health facility. During this second stage of training, all MCH service providers were given an overview of the TextIT implementation protocol, the data collection procedures, SMS-based participant registration, and the role of the mentor mother in implementing TextIT. This second stage also allowed trainers to meet mentor mothers and address any gaps in competency identified by the evaluation following the initial 2-day training.

### Interventions and study procedures

At facilities randomized to continue usual care, pregnant women did not receive any study-related intervention during the first phase of the trial. Mentor mothers at these facilities were trained, at their respective facilities, on abstracting data. They were aware of the general nature of the intervention and outcome being measured but did not receive any other training until the end of phase I of the study, immediately prior to implementing the intervention in the second phase. When health facilities implemented TextIT, both in phase I (intervention facilities only) and phase II (all facilities) of the study, mentor mothers were trained to provide a brief overview of the TextIT strategy, obtain verbal consent to receive the intervention, and provide a written information statement to women willing to receive messages. Baseline clinical and demographic characteristics were recorded only for women who provided verbal informed consent, after which they were registered to receive intervention text messages. Participants attending clinics implementing TextIT continued to receive standard care even if they did not have access to a phone and were considered as having been exposed to the TextIT intervention in the intent-to-treat analysis.

Women registered in the SMS system received up to 14 text messages during pregnancy and after delivery. The message content and schedule were the same as those used in the earlier study demonstrating the efficacy of this intervention [5]. Participants at facilities implementing TextIT had the option to respond to text messages, call, or send inquiry text messages to a designated clinic phone. One nurse was assigned to respond to all calls and text messages. During the intervention period, if delivery had not occurred at the facility, the TextIT nurse called intervention recipients weekly, beginning at 37 weeks’ gestation, to determine whether they had delivered elsewhere. These calls continued until delivery status was ascertained. For both intervention and control periods, if no record of delivery existed at the facility at the end of the follow-up period, clinic staff obtained this information at the first postnatal contact with the mother (for women who visited the clinic).

### Outcomes

Primary outcome measures were infant HIV virologic testing—defined as obtaining a dried blood spot sample for HIV polymerase chain reaction (PCR) testing within 8 weeks after birth—and the proportion of women retained in postpartum care—defined as documented return for at least one visit at the PMTCT, postnatal, or general HIV care clinic within 8 weeks after delivery. We assessed infant HIV testing by abstracting information from medical records, including the HEI follow-up register, HEI card, and laboratory registers. We assessed maternal postpartum retention by abstracting information from patient charts and clinic records. As part of routine care, women who failed to attend clinic appointments were traced to attempt to reengage them in care. We relied on this well-established tracing program to ascertain birth and other outcomes for women who failed to return to clinic [9,10]. Women who could not be contacted were considered to have failed to obtain HIV testing for their infants. Women who voluntarily transferred out to facilities not supported by FACES were excluded from analysis, because we had no mechanism to trace them outside the FACES network. For pregnancies resulting in more than one infant, our analysis was restricted to the firstborn infant. Infants of women who died during follow-up, stillbirths (fetal loss after 28 weeks of pregnancy) and infant deaths before 8 weeks were excluded from the analysis.

### Power and sample size determination

The primary outcome for sample size determination was infant HIV virologic testing. This outcome was binary and measured at the individual level. We estimated a relative risk comparing the proportion of infants who were tested for HIV in the intervention period compared to proportion tested within the control period. Power was determined using the method described by Hussey and Hughes [11]. We assumed a coefficient of variation between clusters of 0.25 and calculated the power for a two-tailed test with α = 0.05. Assuming that 40% of infants in the control group would undergo HIV testing (based on observed testing rates within the Nyanza region prior to the TextIT study), with 20 clusters and 2 time points, we estimated that a harmonic mean of 57 individuals per cluster per time interval would achieve ≥90% power to detect an increase to 53% tested or greater in the intervention group. Presuming 10% loss to follow-up, we proposed a final sample size of 2,508 women at 20 clinics overall.

### Statistical analysis

All analyses were performed using Stata software (StataCorp, College Station, TX). We described participant characteristics at baseline in control and intervention groups. Inferential analysis for the primary outcomes followed the intent-to-treat principle. The predictor of interest was treatment assignment at the clinic/cluster level (i.e., intervention versus control clinics). Time period and stratum of randomization were included as covariates [12]. Our analysis used modified Poisson regression with robust variance estimation to estimate the relative risk and 95% confidence intervals (CIs). We applied generalized estimating equations on individual-level data with working exchangeable correlation structure to account for clustering by site [13]. All tests were two-sided and conducted at the 5% significance level.

### Secondary analyses

We conducted secondary analyses to better understand potential changes in the effect of the intervention over time. We also explored whether adjustment for time period resulted in smaller effect sizes due to infant testing and maternal retention increasing over time, independent of the intervention. First, we analyzed the intervention effect moving from period 1 to period 2 in the 10 control clusters that were not assigned to begin receiving the intervention until period 2 (before-after analysis). Second, we analyzed the intervention effect in period 1 alone, comparing infant testing and maternal retention between intervention and control clusters (equivalent to analysis of a parallel cluster randomized study). Finally, we analyzed the change in outcome proportions between period 1 and period 2 in the clusters assigned to begin receiving the intervention in period 1 (intervention clusters). This analysis would allow us to identify a change in outcomes over time in the presence of a consistent intervention, which could be due to a secular trend or a time-dependent change in the intervention effect.

### Ethical considerations

County health officials provided written permission for the health facilities in their respective jurisdictions to take part in the study. All text message recipients provided verbal informed consent to receive the messages. For women at health facilities not implementing the TextIT intervention, we had approval from the relevant institutional review boards to collect study data from clinical data that were already being captured routinely for evaluation without obtaining individual-level consent. Ethical approval was obtained from the Kenya Medical Research Institute’s Scientific and Ethics Review Unit, the University of Washington’s Human Subjects Division, and UCSF’s Committee on Human Research. The trial is registered in ClinicalTrials.gov (NCT02350140).

## Results

### Study participants

**Fig 2:**
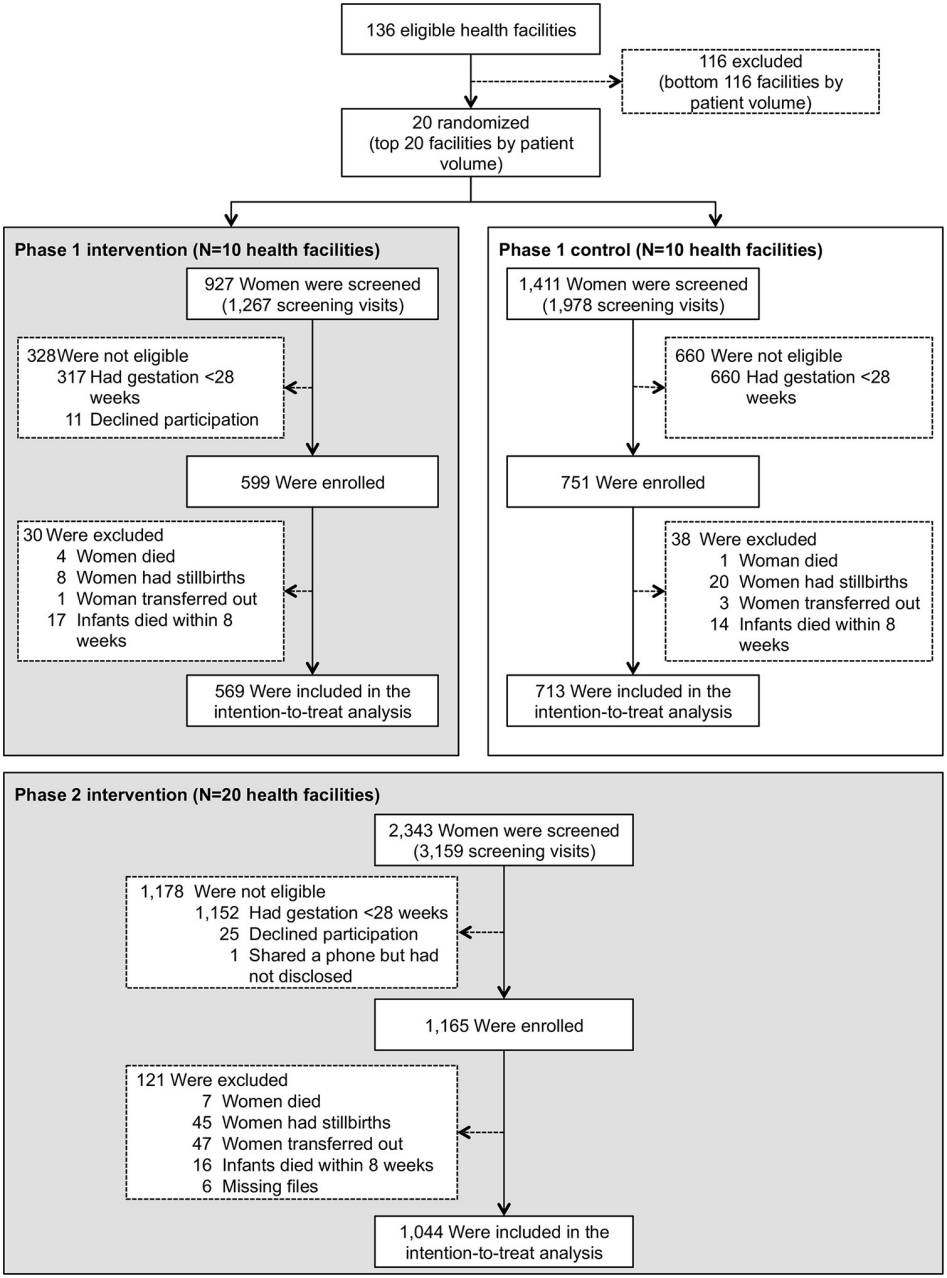
Study profile. Gray background represents intervention periods; white background represents control period.

The flow of clusters and participants through the study is shown in Fig 2. Between February 2015 and December 2016, 4,681 women were assessed for study participation at 20 health facilities. Of these, 2,129 who were less than 28 weeks pregnant were excluded. In the intervention phases, we excluded 36 women who declined participation and 1 who shared a phone but had not disclosed her HIV status to the person sharing. Overall, 2,515 women participated in the study, including 751 at phase I control facilities, 599 at phase I intervention facilities, and 1,165 in phase II across all 20 facilities.

Table 1 shows baseline characteristics of the study clinics. Table 2 compares the baseline characteristics of women by intervention (SMS) and control groups, and Table 3 compares baseline characteristics of infants in intervention and control groups. Maternal demographic and clinical characteristics were well balanced across intervention and control groups. Overall, the median age was 27 years (interquartile range [IQR] 23–30). The median gestational age was 30 weeks (IQR 28–34). The majority of women were unemployed (1,916/2,515; 76%), had completed primary education (2,213/2,515; 78%), and were married (2,297/2,515; 91%). The median CD4 cell count at enrollment was 479 cells/μL (IQR 329–663), and nearly all women (2,487/2,515; 99%) were receiving ART. Among the 1,764 women enrolled during intervention phases, 1,534 (87%) owned a phone. Of these, 470 (31%) reported sharing their phone and had disclosed their HIV status to the person sharing. The primary analysis of infant HIV testing included 2,326 infants, while the primary analysis of maternal retention in postpartum care within 8 weeks after delivery included 2,472 women. The perinatal mortality rate in this cohort, which reflects the combined number of stillbirths plus early infant deaths, was 44 per 1,000 total births.

**Table 1:** Health facility characteristics.

Characteristic	Intervention phase I *N* = 10 Median (IQR) or number (%)	Intervention phase II *N* = 10 Median (IQR) or number (%)
**Level of facility**		
Tertiary-level hospital	3 (30%)	3 (30%)
Secondary-level hospital	2 (20%)	1 (10%)
Primary level (health center or dispensary)	5 (50%)	6 (60%)
**Number of health workers by cadre**		
Mentor mothers	2 (1–5)	2 (2–2)
Nurses	9 (4–25)	7 (4–19)
Clinical officers	6 (4–12)	4 (3–12)
Lab technologists	3 (1–8)	2 (2–8)
HIV testing and counseling officers	3 (2–5)	3 (2–4)
Pharmacy technicians	3 (1–5)	2 (1–6)
Medical doctors	1 (0–3)	0 (0–1)
Clinic and community health assistants	2 (1–3)	2 (2–3)
Peer educators	1 (1–4)	3 (2–4)
Health records and information officers	1 (1–3)	1 (1–2)
Patient trackers	1 (1–2)	2 (1–3)
Nutritionists	1 (0–2)	1 (0–3)
Physiotherapists	0 (0–0)	0 (0–2)
**Managing authority**		
County	9 (90%)	10 (100%)
Nongovernmental organization	1 (10%)	0 (0%)
**Urban location (versus rural)**	4 (40%)	4 (40%)
**Total catchment population**	10,722 (9,012–25,366)	11,233 (7,908–18,318)

**Abbreviation:** IQR, interquartile range

**Table 2:** Maternal baseline demographic and clinical characteristics.

Characteristic	SMS (*N* = 1,764)	Control (*N* = 751)
	*N* (%)	*N* (%)
**Maternal age (y)**		
<18	29 (1.6)	17 (2.3)
18–24	569 (32.3)	257 (34.2)
25–34	983 (55.7)	406 (54.1)
35+	183 (10.4)	71 (9.5)
**Employed**	388 (22)	211 (28.1)
**Education**		
None	248 (14.1)	54 (7.2)
Primary	1,113 (63.1)	536 (71.4)
Secondary	321 (18.2)	138 (18.4)
Post-secondary	82 (4.6)	23 (3.1)
**Luo ethnicity (versus other)**	1,673 (94.8)	710 (94.5)
**Married**	1,600 (90.7)	697 (92.8)
**First pregnancy**	171 (9.7)	86 (11.5)
**No previous deliveries**	170 (9.6)	86 (11.5)
**WHO clinical stage**		
I	1,128 (63.9)	489 (65.1)
II	410 (23.2)	166 (22.1)
III	193 (10.9)	78 (10.4)
IV	33 (1.9)	18 (2.4)
**Most recent CD4 cell count (cells/μL)**		
<200	143 (8.1)	61 (8.1)
200–349	303 (17.2)	152 (20.2)
350–500	390 (22.1)	180 (24)
500+	775 (43.9)	311 (41.4)
**Receiving ART**	1,740 (98.6)	747 (99.5)
**Nevirapine prophylaxis for baby issued**	1,759 (99.7)	751 (100)
**HIV test done on screening day (versus earlier)**	90 (5.1)	64 (8.5)
**HIV counseling with partner**	580 (32.9)	279 (37.2)

**Abbreviations:** ART, antiretroviral therapy; SMS, short message service

**Table 3:** Infant characteristics at birth.

Characteristic	SMS (*N* = 1,684)	Control (*N* = 695)
	*N* (%)	*N* (%)
**Gestational age at delivery (wk), median IQR**	39 (37–41)	39 (36–41)
**Live births (versus stillbirths)**	1,629 (96.7)	675 (97.1)
**Female**	842 (47.7)	328 (43.7)
**Birth weight (kg), median (IQR)**	3.2 (3.0–3.5)	3.2 (3.0–3.5)
**Delivery at health facility (versus home)**	1,508 (85.5)	621 (82.7)
**Delivery by cesarean section**	56 (3.2)	18 (2.4)
**Exclusive breastfeeding (versus other)**	1,577 (89.4)	653 (87)

**Abbreviations:** IQR, interquartile range; SMS, short message service

Of the 2,326 infants analyzed, 1,466 of 1,613 (90.9%) in the intervention group and 609 of 713 (85.4%) in the control group had HIV virologic tests performed before 8 weeks (adjusted relative risk [aRR] 1.03; 95% CI 0.97–1.10; *P* = 0.3) (Table 4). Of 2,472 women analyzed, 1,548 of 1,725 (90%) in the intervention group and 571 of 747 (76%) in the control group were retained in care during the first 8 weeks after delivery (aRR 1.12; 95% CI 0.97–1.30; *P* = 0.1) (Table 4).

**Table 4:** Unadjusted and adjusted analyses of effect of SMS on infant HIV testing and maternal postpartum retention.

Outcome	SMS	Control	Unadjusted RR (95% CI)	*P* value	aRR^a^ (95% CI)	*P* value
**Infant HIV testing**	1,466/1,613 (90.9%)	609/713 (85.4%)	1.07 (1.02–1.11)	0.002	1.03 (0.97–1.10)	0.3
**Maternal postpartum retention**	1,548/1,725 (89.7%)	571/747 (76.4%)	1.18 (1.03–1.34)	0.01	1.12 (0.97–1.30)	0.1

^a^Adjusted for intervention time period and randomization stratum; control group as reference.

**Abbreviations:** aRR, adjusted relative risk; CI, confidence interval; RR, relative risk; SMS, short message service

### Secondary analyses

In the before-after analysis of the effect of the intervention moving from period 1 to period 2 among the 10 clusters that were assigned to not receive the intervention until period 2, 85% of infants had received HIV testing in period 1 compared to 91% who received testing in period 2 (aRR 1.07; 95% CI 1.03–1.11; *P* = 0.002; Table 5), and 77% of women were retained in care in period 1 compared to 90% in period 2 (aRR 1.18; 95% CI 1.03–1.35; *P* = 0.02 Table 6). When the analysis comparing intervention and control clusters was restricted to period 1 alone, there were no statistically significant differences in infant HIV testing (aRR 1.04; 95% CI 0.98–1.11; *P* = 0.2) or maternal retention (aRR 1.12; 95% CI 0.97–1.30; *P* = 0.1) outcomes. Among clusters assigned to receive the intervention starting in period 1, there were no significant differences in the proportions of infants tested (Table 5) or mothers retained post partum (Table 6) comparing period 1 and period 2.

**Table 5:** Secondary analyses of effect of SMS on infant HIV testing.

Cluster	Period 1	Period 2	Intervention effect RR (95% CI)
Control clusters*	609/713 (85.4%)	479/525 (91.2%)	1.07 (1.03–1.11)**
Intervention clusters***	507/569 (89.1%)	480/519 (92.5%)	N/A
**Intervention effect RR (95% CI)**	1.04 (0.98–1.11)**	N/A	N/A

*Control clusters refer to the 10 clinics assigned to begin receiving SMS in period 2 (control).

**Adjusted for randomization stratum; exposure is intervention (versus control).

***Intervention clusters refer to the 10 clinics assigned to begin receiving SMS in period 1 (intervention).

**Abbreviations:** CI, confidence interval; RR, relative risk; SMS, short message service

**Table 6:** Secondary analyses of effect of SMS on maternal postpartum retention.

Cluster	Period 1	Period 2	Intervention effect RR (95% CI)
Control clusters*	571/747 (76.5%)	507/565 (89.7%)	1.18 (1.03–1.35)**
Intervention clusters***	526/596 (88.3%)	515/564 (91.3%)	N/A
**Intervention effect RR (95% CI)**	1.12 (0.97–1.30)**	N/A	N/A

*Control clusters refer to the 10 clinics assigned to begin receiving SMS in period 2 (control).

**Adjusted for randomization stratum; exposure is intervention (versus control).

***Intervention clusters refer to the 10 clinics assigned to begin receiving SMS in period 1 (intervention).

**Abbreviations:** CI, confidence interval; RR, relative risk; SMS, short message service

## Discussion

In this pragmatic, cluster-randomized, stepped-wedge trial of a theory-based text messaging intervention to improve uptake of infant HIV testing under routine care conditions, a greater proportion of infants of women in the intervention group received HIV testing compared with the standard care group, but the difference was small and not statistically significant. There was also a nonsignificant increase in postpartum retention of mothers in the intervention periods compared to control.

It is encouraging that the proportions of infants tested and mothers retained in the intervention group of the present effectiveness study were high (91% and 90%, respectively), especially given that 13% of women in the intervention group did not have phone access, so they were exposed to the intervention only in the context of their facilities’ participation. In the earlier efficacy trial, women without phones were ineligible [5].

The findings in the present study were likely influenced by high rates of early infant HIV testing (85%) and maternal retention in postpartum care (76%) even during the control period in facilities that did not receive the intervention during the first phase of the trial. The high proportion of early infant HIV testing and maternal retention in the control group could be due to several factors. First, “contamination” with usual care procedures could have occurred in control facilities. Health workers at control facilities were aware of ongoing monitoring for the trial, including evaluation of rates of EID completion, and could have made the extra effort to counsel women about the importance of infant HIV testing. Facility staff might also have followed up women in the PMTCT program more keenly because of their knowledge of the monitoring, akin to the Hawthorne effect. Second, there were concerted efforts by programs in Kenya, and FACES in particular, aimed at increasing EID to meet the deadline for eliminating mother-to-child HIV transmission by end of 2015 [14]. Many of these interventions were implemented around the same time as the present trial [15]. In addition, FACES modified defaulter management procedures by including phone calls to women who missed clinic appointments, including those for EID. Finally, introduction of the Kenya Mentor Mother Program could have contributed to improvements [8]. An integrated package of similar interventions significantly improved maternal retention and EID rates in a randomized trial in Nigeria [16].

We found that the perinatal mortality rate in this cohort of HIV-infected women (44 deaths per 1,000 total births) was 1.5 times higher than the reported national rate of 29 deaths per 1,000 total births [17]. This finding suggests that there may be an additional gap in the PMTCT continuum and highlights the need for further effort to ensure safe delivery services for women living with HIV.

Our study had a number of limitations. First, awareness of ongoing observation and evaluation of EID completion rates at control facilities could have resulted in bias towards higher EID at control facilities and a null effect of the trial. In addition, some facilities in the control condition had been part of a pilot implementation of the intervention prior to the effectiveness study. This limitation was mitigated, but not eliminated, by stratifying randomization according to prior experience with the intervention, as described in the randomization section of this manuscript. Second, our study was conducted in a high–HIV-prevalence area. In addition, all health facilities that provide HIV services in this region also receive considerable support from HIV/AIDS care and treatment programs. The high HIV prevalence as well as presumably higher level of care than at facilities not receiving external support could reduce the generalizability of our findings to areas with low HIV prevalence or to clinics outside this region that may not receive external support for HIV services. Finally, it was not possible to distinguish a real secular trend from an apparent trend caused by improvement in the intervention over time with this study design. To examine this effect directly would have required a third control arm that did not receive the intervention during the entire course of the trial. Importantly, our secondary analyses revealed an increase in infant testing and maternal retention in the clusters that received the intervention in both time periods (although this increase is less than the intervention effect). This could represent a true secular trend over time that would have occurred even if there was no intervention (i.e., improvement in outcomes in the broader community from which the clinics were drawn, completely independent of the intervention). The increases in infant testing and maternal retention in the facilities that were randomized to receive the intervention in both time periods could also be due to improvement over time in the intervention effect (i.e., a learning effect whereby improvement occurs with prolonged use and gain in experience).

Koepsell and colleagues note that interventions that show improvement in efficacy trials do not always yield the same results when tested more broadly [18]. An instructive example is the failure to replicate “model” substance abuse programs in the real world, despite evidence of efficacy from smaller randomized trials [19]. To our knowledge, our study is the first in the field of implementation science for HIV to demonstrate the feasibility (and pitfalls) of moving an evidence-based mHealth intervention from the controlled environment of a randomized trial to the complex real world of implementation to achieve “relevance without sacrificing rigor,” as recently articulated in an Editorial in *PLOS Medicine* [20]. Our study highlights the importance of conducting effectiveness studies as part of the continuum of implementation to confirm that efficacy trial results can be replicated with fidelity under complex real-world contexts. The low cost of this intervention makes it a “low-hanging fruit” that PMTCT programs globally could easily adopt and disseminate more broadly. For example, a single nurse in the present trial’s study team performed tasks essential to intervention delivery, including responding to phone calls and messages from women receiving the intervention, calling women to determine delivery status, and training mentor mothers. The cost of incoming and outgoing text messages and calls was approximately 25 Kenya shillings per woman (approximately 0.25 USD) over the entire study period. Initial setup costs were estimated at about USD 7,000 and included the costs of setting up and hosting the automated text messaging software, training sessions for mentor mothers, buying phones for each of the study sites, and providing mobile phone connectivity for data transmission between study sites and the central “server” phone.

Notably, while the *P* values reported for the primary analyses in this pragmatic effectiveness trial do not meet the conventional significance threshold of 0.05, there may be value in considering the results in their totality, with attention to the 95% CIs and to the evidence from the earlier efficacy trial. For example, the 95% CIs around the aRRs range from a 3% decreased probability to a 10% increased probability of infant HIV testing, and from a 3% decreased probability to a 30% increased probability of being retained in care. In the context of this pragmatic implementation trial of a low-risk and low-cost intervention that was previously shown to be significantly efficacious, it may be most appropriate to interpret these results based on the extent to which the data are compatible or incompatible with an effect similar to that observed in the earlier trial.

In the context of conducting pragmatic implementation research, this study illustrates the difficulty of evaluating intervention effectiveness against a backdrop of what may already be an improving system. Ongoing programmatic efforts to improve retention in PMTCT likely contributed to the rise in background retention rates that were observed in this pragmatic implementation effectiveness trial compared to our earlier efficacy trial of the TextIT intervention. Within the FACES program, these included a series of “rapid results initiative” interventions to improve uptake of PMTCT services through simple but intensive strengthening of existing health systems [21], integration of PMTCT services with antenatal care [15], and initiatives to increase male partner involvement in PMTCT [22]. What remains unknown is how the intervention would have performed in a setting that started out with low infant testing and maternal retention rates. Under such conditions, it might be worth considering having a control group that does not receive the intervention during analysis, and could still receive the intervention after analysis, especially if there is a chance that the system is improving independent of the intervention.

The high rates of EID during the control period in the delayed intervention arm in this study provide indirect evidence that simple improvements in service delivery (such as staff awareness of monitoring) could increase EID rates substantially. Despite the lack of a significant effect of the intervention on EID rates and maternal retention in this study, key lessons have emerged, both for strengthening efforts to eliminate mother-to-child HIV transmission and for implementation research more generally. Perhaps most important, improving implementation during the control period in delayed intervention facilities, primarily through staff awareness of monitoring, was sufficient to substantially improve infant HIV testing rates. Our earlier efficacy study showed that under ideal conditions, text messages could have a benefit beyond good service delivery. In contrast, the present study showed that in a real-world setting, most of the benefit was probably from the basic improvements created by monitoring. Nonetheless, we feel that this intervention could be important in the context of both low and high rates of EID and maternal follow-up in PMTCT. In both contexts, it would likely be implemented along with other ongoing programmatic activities designed to improve delivery of existing services. In settings in which EID and maternal follow-up outcomes are poor, the observed effect of TextIT is likely to be larger. In settings in which EID and maternal follow-up outcomes are already improving, TextIT may provide the boost needed to reach the Joint United Nations Programme on HIV/AIDS (UNAIDS) 90-90-90 targets.

## Supporting information

S1 Trial protocol (PDF)

S1 CONSORT Checklist (DOCX)

## Abbreviations

aRR: adjusted relative risk
ART: antiretroviral therapy
CDC: Centers for Disease Control and Prevention
CI: confidence interval
EID: early infant diagnosis
FACES: Family AIDS Care and Educational Services
HEI: HIV-exposed infant
IQR: interquartile range
MCH: maternal and child health
mHealth: mobile technology for health
PCR: polymerase chain reaction
PEPFAR: United States President’s Emergency Plan for AIDS Relief
PMTCT: prevention of mother-to-child HIV transmission
SMS: short message service
UNAIDS: Joint United Nations Programme on HIV/AIDS
