# Supplementary material for: Text messaging for maternal and infant retention in prevention of mother-to-child HIV transmission services: A pragmatic stepped-wedge cluster-randomized trial in Kenya
Source: PLoS Med. 2019 Oct 2;16(10):e1002924. doi: 10.1371/journal.pmed.1002924 (PMC6774469; doi:10.1371/journal.pmed.1002924)
Supplement: S1 Trial protocol — (PDF) [file pmed.1002924.s001.pdf]

1.0 TITLE OF THE PROJECT

Texting to Improve Testing (TextIT): A Cluster Randomized Stepped Wedge Trial of Text Messaging to Improve Postpartum Retention in Care and Early Infant Diagnosis of HIV

2.0 INVESTIGATORS AND INSTITUTIONAL AFFILIATIONS

Principal Investigator:	Thomas A. Odeny, MBChB, MPH, PhD<br>Research Scientist<br>Center for Microbiology Research, KEMRI
Co-Investigators:	Elizabeth Bukusi, MBChB, M.Med, MPH, PhD, PGD<br>Chief Research Officer and Deputy Director (Research and Training)<br>KEMRI<br><br>Co-Director Research Care Training Program<br>Center for Microbiology Research, KEMRI
	R. Scott McClelland, MD, MPH<br>Professor of Medicine, Epidemiology, and Global Health<br>University of Washington
	Craig R. Cohen, MD, MPH<br>Professor<br>Department of Obstetrics, Gynecology and Reproductive Sciences<br>University of California, San Francisco
	Eliud Akama, BSN<br>Provincial Technical Advisor – PMTCT<br>Family AIDS Care and Educational Services (FACES)

TABLE OF CONTENTS

1.0 TITLE OF THE PROJECT 1

2.0 INVESTIGATORS AND INSTITUTIONAL AFFILIATIONS 1

3.0 ABSTRACT 4

4.0 BACKGROUND 6

5.0 JUSTIFICATION 8

6.0 HYPOTHESES 10

7.0 STUDY OBJECTIVES 10

7.1 GENERAL OBJECTIVE 10

7.2 SPECIFIC OBJECTIVES 10

7.3 SECONDARY OBJECTIVES 10

8.0 DESIGN AND METHODOLOGY 11

- 8.1 Study Design 11
- 8.2 Study site 12
- 8.3 Study populations 12
- 8.4 Randomization Strategy and Sequence generation 13
- 8.5 Allocation concealment 14
- 8.6 Implementation of randomization procedures 14
- 8.7 Blinding 14
- 8.8 Sample size determination 14
- 8.9 STUDY PROCEDURES 15

9.0 ETHICAL CONSIDERATIONS 18

- 9.1 Informed Consent 18
- 9.2 Ethical Approval 18
- 9.3 Protecting privacy and confidentiality 19
- 9.4 Potential risks of proposed research to study subjects 19
- 9.5 Potential benefits of proposed research to study subjects and others 19

10.0 DATA MANAGEMENT 20

10.1 Data Collection 20
10.2 Data Storage and Security 20
11.0 TIME FRAME 23
12.0 EXPECTED APPLICATION OF THE RESULTS 23
13.0 REFERENCES 24
14.0 APPENDICES 27
14.1 Participant Information Statement – English 27
14.2 Participant Information Statement – Kiswahili 27
14.3 Participant Information Statement – Dholuo 27
14.4 Baseline questionnaire 28
14.5 Postnatal visit questionnaire 31
14.6 Table of intervention text messages 33

3.0 ABSTRACT

Early accurate diagnosis is one of the first crucial steps in care for infants born to HIV-infected mothers. Early initiation of antiretroviral therapy (ART) relies upon early diagnosis and results in significant reductions in infant morbidity and mortality. We recently concluded a successful randomized controlled trial (RCT) in Kenya entitled, "Improving uptake of early infant diagnosis of HIV for PMTCT: a randomized trial of a text messaging intervention" (ClinicalTrials.gov # NCT01433185; KEMRI SSC No. 2103). In this study, text messages developed using a behavioral theoretical framework significantly improved maternal attendance at post-partum clinic appointments and rates of testing to facilitate early infant diagnosis of HIV in a selected population and controlled setting. Understanding the effectiveness of this intervention (and its limitations) in a real-world, routine-care setting represents the next step in the translational pathway to public health impact. We therefore now propose a cluster randomized, stepped wedge trial in 20 clinics operated by the Kenyan Ministry of Health in the Nyanza region of Kenya and use the REAIM framework to understand the effectiveness of the intervention: **R**each, **E**ffectiveness, **A**doption, **I**mplementation, and **M**aintenance (the RE-AIM framework). Our specific aims are:

1. To determine the effect of TextIT on maternal attendance at postpartum clinic visits during the randomized stepped-wedge rollout of the intervention.

   Hypothesis 1: A greater proportion of women at health facilities implementing TextIT will attend clinic within eight weeks postpartum compared to women at health facilities implementing standard care.

2. To determine the effect of TextIT on virological infant HIV testing within eight weeks after birth during the randomized stepped-wedge rollout of the intervention.

   Hypothesis 2: Infants of women at health facilities implementing TextIT will be more likely to have virological HIV testing compared to infants of women at health facilities implementing standard care.

3. To determine the costs and cost-effectiveness of TextIT. We will estimate the cost per patient and per health gain achieved (disability-adjusted life year, DALY) comparing TextIT to current standard care.

   Hypothesis 3: The TextIT intervention will be more cost-effective than current standard care.

We will conduct additional ancillary studies to assess the success of implementation. Assessing implementation outcomes will allow us to determine not only the effectiveness of the intervention in a real world, routine care setting, but also the extent to which the intervention is implemented with fidelity to the original design. This will include using the RE-AIM framework to determine the overall public health impact of TextIT.

LIST OF ACRONYMS

AE	Adverse Experience
AIDS	Acquired Immunodeficiency Syndrome
ANC	Ante-natal Clinic
ARV	Antiretroviral
CDC	Centers for Disease Control
CI	Confidence Interval
CMR	Center for Microbiology Research
CONSORT	Consolidated Standards of Reporting Trials
DNA	Deoxyribonucleic Acid
ERC	Ethical Review Committee
FACES	Family AIDS Care and Education Services
HBM	Health Belief Model
HIV	Human Immunodeficiency Virus
ID	Identification number
IRB	Institutional Review Board
KEMRI	Kenya Medical Research Institute
MCH	Maternal and Child Health
MOH	Ministry of Health
NIH	National Institutes of Health
PEPFAR	President’s Emergency Plan for AIDS Relief
PI	Principal Investigator
PCR	Polymerase Chain Reaction
PMTCT	Prevention of Mother-to-Child Transmission
PNC	Post-natal Clinic
SMS	Short Messaging Service
STD	Sexually Transmitted Disease
UCSF	University of California, San Francisco
UN	United Nations
UNICEF	United Nations Children’s Fund
UNAIDS	Joint United Nations Program on AIDS
WHO	World Health Organization

4.0 BACKGROUND

The 2011 United Nations General Assembly Political Declaration on HIV/AIDS set a target to eliminate mother-to-child transmission of HIV by 2015 [2]. However, access to services for prevention of mother-to-child transmission of HIV (PMTCT) in sub-Saharan Africa, where 92% of pregnant women living with HIV reside, is still low. Currently, 59% of women have access to these services, compared to the 80% target set by the 2011 United Nations General Assembly Special Session on HIV/AIDS (UNGASS). Mother-to-child HIV transmission rates remain high, ranging from 17% in southern Africa to about 30% in central and western Africa [3].

Retention of mother-child pairs in the postpartum period

The continuum of care for women living with HIV and their babies includes retention in PMTCT programs and early infant diagnosis (EID) of HIV. Timely initiation of infant antiretroviral therapy (ART) requires HIV-positive women to be retained in PMTCT care through the postpartum period and to bring their children for HIV testing. However, a high proportion of pregnant HIV-positive women in sub-Saharan Africa are lost to follow-up after delivery. For example, in Nigeria, a large ART program reported that only 52% of women who began PMTCT care in the antenatal period had at least one follow-up visit after delivery [4]. Low adherence to postnatal PMTCT has also been reported by programs in Uganda (38% at eight weeks) [5], Zimbabwe (14.8% at six weeks) [6], and Ethiopia (10.6% of HIV positive women had their children tested) [7]. The World Health Organization (WHO) recommends infant HIV testing at six weeks using DNA polymerase chain reaction (PCR) [8]. However, the overall proportion of children who undergo EID by PCR remains low. Only 28% of infants born to HIV-positive women in low- and middle-income countries in 2010 were tested for HIV within two months of birth [9]. In sub-Saharan Africa, PMTCT programs report widely varying proportions of early infant diagnosis, ranging from 25% in Mozambique to between 54% and 72% in Malawi [10-12]. In Kenya, the overall proportion of eligible children undergoing PCR testing in 2011 was 39% [3].

Low rates of postpartum PMTCT retention and infant HIV testing in many sub-Saharan African countries pose a major threat to successful completion of the PMTCT cascade of care. Low rates of infant HIV testing are an indirect indication of a large number of infants who may not benefit from early determination of HIV status, antiretroviral prophylaxis for HIV-negative breastfeeding infants, and infant feeding counselling and support. For HIV positive infants, failure to undergo testing is a critical barrier to receiving life-saving ART. Among children who acquire HIV infection in the peri-partum period, more than half die within one year in the absence of ART [13].

There is an urgent need to investigate efficacious, cost-effective, and sustainable interventions to improve maternal retention in PMTCT and increase the proportion of exposed infants tested for HIV. Strategies might include interventions that are associated with improved retention in PMTCT such as improved interaction with providers [14], improving the knowledge of mothers about benefits of attending postnatal PMTCT clinics [5, 15], and use of phone reminders [5].

Mobile phone technology to support HIV interventions (mHealth)
With the exponential increase in the number of mobile phones in sub-Saharan Africa, the use of mobile technology to support HIV programs has shown promise. In Uganda, a mobile health (mHealth) program that provided community health workers with mobile phones for communicating patient information to a central clinic suggested that such interventions are supported by both patients and providers [16]. In Malawi, a similar intervention resulted in cost savings [17].

In PMTCT programs, mHealth has been successfully used to reduce the time between sample collection for infant testing and result notification [18]. Access to a mobile phone is significantly associated with postpartum retention in PMTCT [5], and reminder calls from health workers may result in increased return for clinic appointments [19]. Reminder phone calls paired with home visits may be efficacious in improving early infant diagnosis of HIV [20, 21]. In one study in South Africa, SMS reminders (compared to no SMS) were associated with a significant increase in the proportion of infants tested for HIV at six weeks [22]. The United Nations Children's Fund (UNICEF) is encouraging PMTCT programs to take advantage of high levels of mobile phone access among enrolled mothers by reminding them to return for critical appointments [19]. Active follow-up of PMTCT clients in resource-limited settings using mobile phones is feasible, and may be effective in improving postnatal PMTCT retention and uptake of EID across different sub-Saharan African settings [20, 21].

Preliminary research
The Kenya Strategic Framework for Elimination of Mother-to-Child Transmission of HIV 2012-2015 lists priority research questions that include: how technology can be used to optimize care for mother-baby pairs, and how mobile phones can be used to improve retention in HIV care [23]. We developed an interactive two-way text messaging system using a behavioural theoretical framework, then demonstrated the efficacy of our messages for improving rates of maternal postpartum clinic attendance and early infant HIV testing by PCR. We conducted a two-phase study, beginning with formative qualitative research to develop the intervention text messages. We conducted five focus group discussions (FGD) with health workers and women attending antenatal, postnatal, and PMTCT clinics. We used the Health Belief Model as the theoretical framework for facilitating the FGDs and crafting the intervention text messages. We developed a set of messages in local languages that were personalized by having an option to insert the mothers' and infants' names. A manuscript reporting this theory-based message development process is undergoing peer review.

In the second phase, we conducted a parallel-group, unblinded, randomized controlled trial in western Kenya to evaluate the efficacy of our messages for improving post-natal clinic attendance and early infant HIV testing (SMS4PMTCT Study). Participants were recruited from among women attending antenatal care (ANC) clinics at five health facilities supported by the Family AIDS Care and Education Services (FACES). FACES is a PEPFAR/CDC funded HIV prevention, care and treatment program operated jointly by the Kenya Medical Research Institute (KEMRI) and the University of California, San Francisco. FACES cares for over 80,000 patients and supports 132 government health facilities spread across three counties in the Nyanza region, western Kenya, to offer PMTCT services. This region has the highest HIV

prevalence in Kenya (15%). The HIV prevalence is even higher among pregnant women (18%) [24, 25]. The five health facilities were a mix of rural and urban health facilities.

Participants were eligible for study enrolment if they were at least 18 years old, reported being able to read SMS or had someone who usually read SMS on their behalf, were between 28 weeks gestation and the day of delivery, were enrolled in the PMTCT program, were planning to remain in the study area for the duration of the study, had access to a mobile phone, and were willing to receive SMS messages from the study. Women who reported sharing phones were enrolled only if they had disclosed their HIV status to the person with whom the phone was shared.

We randomized 388 HIV positive pregnant women to either receive SMS or usual standard of care. In the SMS group, 172/187 (92.0%) infants were tested within the recommended eight weeks compared to 154/181 (85.1%) in the control group (Relative risk [RR] 1.08; 95% CI 1.00 to 1.16; p=0.04). Women in the SMS group were also significantly more likely to attend a post-partum clinic visit compared to those in the control group (38/194, 19.6% versus 22/187, 11.8%; RR 1.66, 95% CI 1.02 to 2.70; p=0.04).

In this study, we achieved >90% infant testing within the recommended eight weeks in the intervention arm. This was significantly higher than in the control arm with no SMS, and was a huge improvement over rates of follow up in observational studies of the same population (37%). This SMS intervention has the potential to greatly improve rates of early infant diagnosis, providing a cost saving, cost-effective and sustainable way of achieving the first crucial step in linking these infants to life-saving treatment. For women, increased rates of PMTCT clinic attendance provide an opportunity for receiving counseling on infant feeding options, access to family planning services, counseling on danger signs, and assessment for eligibility for antiretroviral therapy.

5.0 JUSTIFICATION

Few published studies provide strong evidence of the impact of mobile phone technologies to improve postpartum retention in PMTCT programs and early infant diagnosis of HIV. Furthermore, where studies have used text messaging targeted at behaviour change to improve certain aspects of PMTCT, messages generally have not been based on health behaviour theories that facilitate replication in other settings [26].

A series of recent publications in PLOS Medicine on mHealth report that the mechanisms of action of published mHealth interventions are not adequately described [27]. This series also suggests that mHealth applications may require "theoretical and qualitative approaches" to enable an understanding as to why they may be effective [28], and that many mHealth interventions lack a foundation on sound behavioral theory [26]. Importantly, Tomlinson et al. noted that expanding mHealth interventions that lack a theoretical framework could lead to wastage of resources because such projects would likely not succeed [26]. To our knowledge, our study is among the first mHealth studies in the field of HIV prevention to develop theory-based text messages, evaluate the messages using a rigorous randomized trial design, and report positive results.

The Society for Prevention Research has developed standards to determine the readiness of prevention interventions for scale-up [29]. These standards include efficacy trials under ideal conditions, subsequent effectiveness trials under real-life conditions, and dissemination research to ensure that the intervention can be implemented with “high fidelity” to the design that was tested. Further, they propose that dissemination research should provide information about the intervention's cost and develop tools for monitoring and evaluation.

Evidence of Efficacy

Our study design enabled the establishment of a causal effect between text messaging and improved maternal attendance at postpartum PMTCT clinic and infant HIV testing. Because the duration for measuring these outcomes is fixed, our study had adequate follow-up. Moreover, the effect of this intervention would not be expected to “decay” later on. Therefore, we consider it an example of a high quality **efficacy** study.

Evidence of Effectiveness

We believe that our study also provides preliminary evidence of **effectiveness** because many elements of the trial approximate real-world conditions. For example, we did not have stringent eligibility criteria, so study participants were a close representation of the real-world target population (pregnant HIV-positive women in the third trimester who have access to a mobile phone); study staff were community health workers and peer educators who incorporated study-specific procedures into their daily tasks; our data were abstracted from routine patient charts and clinic registers; and we did not compensate participants for study participation. We developed an *a priori* causal model that included our theory-based intervention – this allowed us to clearly explain the intervention’s causal mechanism. We also developed a text messaging protocol, training manuals, implementation procedures and checklists, data collection forms, lists of frequently-asked questions, and the study software. These features are well suited to allow PMTCT programs to implement the intervention independently and with high fidelity to our model. FACES has already adapted and incorporated our material in a pilot extension to an additional health facility beyond the original five study sites.

Dissemination and implementation science

Given the urgent goal of elimination of mother-to-child transmission of HIV by 2015, we propose to begin a phased dissemination of this new SMS intervention – the **Texting to Improve Testing (TextIT) strategy** – with the ultimate aim of reaching country-wide scale-up in the shortest time possible. Understanding the effectiveness of this intervention (and its limitations) in a real-world, routine-care setting represents the next step in the translational pathway to public health impact. We therefore now propose a cluster randomized, stepped wedge trial in 20 clinics operated by the Kenyan Ministry of Health in the Nyanza region of Kenya.

In order to provide a strong evidence base for successful implementation of the TextIT strategy, we will conduct additional ancillary studies to assess implementation outcomes [1]. This will include an analysis of costs and cost-effectiveness. Interventions that incorporate technology (eHealth/mHealth) might be more efficiently integrated into practice by following theoretical models of implementation [30]. Furthermore, success in the global response to the HIV/AIDS epidemic might require more focus on implementation science research frameworks [31].

Therefore, we will also apply the RE-AIM framework – **R**each, **E**ffectiveness, **A**doption, **I**mplementation, and **M**aintenance – to measure additional implementation outcomes and to determine the overall public health impact of TextIT [32].

6.0 HYPOTHESES

Hypothesis 1: A greater proportion of women at health facilities implementing the TextIT strategy will attend clinic within eight weeks postpartum compared to women at health facilities implementing standard care.

Hypothesis 2: Infants of women at health facilities implementing the TextIT strategy will be more likely to have virological HIV testing compared to infants of women at health facilities implementing standard care.

Hypothesis 3: The TextIT intervention will be more cost-effective than current standard care.

7.0 STUDY OBJECTIVES

7.1 GENERAL OBJECTIVE

Our overall objective is to improve the continuum of care within PMTCT programs by promoting patient retention and enhancing follow-up care for mother-baby pairs.

7.2 SPECIFIC OBJECTIVES

Objective 1: To determine the effectiveness of the TextIT intervention in improving maternal attendance at postpartum clinic visits in a real world, routine care setting.

Objective 2: To determine the effectiveness of the TextIT intervention on improving the rates of virological infant HIV testing within eight weeks after birth in a real world, routine care setting.

Objective 3: To determine the costs and cost-effectiveness of the TextIT intervention by estimating the cost per patient and per health gain achieved (disability-adjusted life year, DALY) compared to current standard care.

7.3 SECONDARY OBJECTIVES

To determine the overall public health impact of the TextIT strategy using the RE-AIM framework which assesses five dimensions of public health interventions: Reach, Effectiveness, Adoption, Implementation, and Maintenance.

Hypothesis: The TextIT strategy will have a higher individual level impact (calculated as the product of Reach × Effectiveness × Implementation) than current standard care. It will reach a high proportion of women who are representative of the target population, be effective at scale, be adopted by targeted health facilities, be implemented with high fidelity to the original design, and be maintained by being institutionalized as a recommendation for routine PMTCT programs in Kenya.

Additional outcomes to be assessed include, but are not limited to: place of delivery; skilled birth attendance (delivery at a health facility or with a skilled attendant present if not at a health

facility); a combined outcome for stillbirth (after 28 weeks of pregnancy) or infant death within the first two months after a live birth; birth weight; reported infant feeding option; and incidence rate of HIV-1 among infants who undergo virological HIV testing. Follow up for mother-baby pairs in PMTCT programs typically ends at 18 months at which time the child's HIV status is confirmed. Therefore, we also aim to assess outcomes at 18 months, including: discharge from the PMTCT program (if confirmed HIV negative); referral and linkage to the general HIV comprehensive care clinic (if confirmed HIV positive); transfer out to another health facility; lost to follow-up; death; and infant HIV status by antibody testing.

8.0 DESIGN AND METHODOLOGY

8.1 Study Design

This will be a pragmatic, cluster randomized, two step, stepped wedge trial. This design is a modification of a stepped wedge design, in that it involves only two time periods of observation [33]. Each time period will last six months. The TextIT intervention will be implemented sequentially at 20 health facilities over the two time periods. Clusters are defined as health facilities supported by the Family AIDS Care and Educational Services (FACES) to provide PMTCT services. Clusters will be selected *a priori* based on ease of study logistics and the administrative costs of expanding the intervention. Specifically, we will consider the patient volume (number of newly infected HIV-positive pregnant women in the prior six months), level of facility, number of peer educators/community health worker, availability of PMTCT services, availability of HIV testing in the maternity and ANC clinic, and availability of services for dried blood spot (DBS) collection at the facility.

The order in which clusters will be assigned to intervention or control time periods will be determined by computer-based randomization as follows:

1. Half of the health facilities will be randomly allocated to receive the TextIT intervention during the first time period (six months), and the other half to continue with current standard care (first step)

2. After the first time period, the remaining 10 facilities will then also receive the intervention (second step)

It is possible that women who are enrolled during the first time period might not have experienced study outcomes by the end of the period. For example, women might enroll during the control period and deliver during the intervention period. Therefore, women who are not receiving the intervention during the crossover period will continue as initially assigned.

Figure 1: Schematic design of the TextIT study

	Observation months 1 – 6	Observation months 7 – 12
Clusters 1 – 10	○	●
Clusters 11 – 20	●	●
Time period	T1	T2

○ Control period
● TextIT intervention period

8.1.1 Rationale for using the cluster randomized stepped wedge design

Our main objective is to understand the effectiveness of this intervention (and its limitations) on a real-world, routine care setting as a path towards achieving public health impact. Therefore, the cluster (health facility) was chosen as the most appropriate unit of randomization. In addition, because this intervention would eventually be implemented at the health facility level, this design allows us to capture the effects of the health system on implementing the intervention [34], and improves the external validity of our findings. As we have previously shown that this intervention is efficacious [35], equipoise may not be justifiable – this intervention will likely do more good than harm. However, due to logistical constraints (such as training logistics) and limited financial and human resources available to us, it would not be feasible to expand this intervention to all facilities at the same time. The stepped wedge design addresses ethical concerns by ensuring that no health facility is deprived of an intervention that is efficacious and potentially effective. A key strength of this design is that the intervention will be rolled out so that all facilities will eventually receive it. Finally, this design avoids "contamination" of the intervention effect. In an individually randomized design, women who are randomized to receive the intervention could share the messages with those randomized to the control arm at the same health facility, resulting in contamination [36]. The study sites are MOH facilities and so are already geographically spread out by design. In this trial, the health facilities are far enough apart that women going to one facility are unlikely to socialize with women from another facility. Randomization at the cluster level thus serves to enhance internal validity.

8.2 Study site

The study will be conducted at health facilities supported by the Family AIDS Care and Education Services (FACES). FACES is a PEPFAR/CDC funded HIV prevention, care and treatment program operated jointly by the Kenya Medical Research Institute (KEMRI) and the University of California, San Francisco. FACES cares for over 80,000 patients and supports 132 government health facilities spread across three counties in Nyanza province, western Kenya, to offer PMTCT services. This region has the highest HIV prevalence in Kenya (15%) [37]. The clinics for conducting this study will be determined based on the location of ongoing PMTCT studies, patient volume, availability of infrastructure, study logistics, and after discussions with health facility management. These health facilities will be selected to be a representative mix of rural, urban, and peri-urban facilities.

8.3 Study populations

8.3.1 Recruitment of study sites

Health facilities for inclusion into the study will be selected by an advisory group composed of: 1) the investigators; 2) two PMTCT experts from FACES; 3) a biostatistician; 4) representatives from the target health facilities or the respective County Health Management Teams; and 5) a community health worker. The investigators will recruit potential study sites by sending letters to the responsible authorities at the selected health facilities. The letter will inform the responsible authorities of the trial's purpose and will include a request for a formal letter agreeing to be part of the study. An accompanying letter of support from the respective County Health Management Teams will also be sought to be sent to the responsible authorities at all selected health

facilities. Through this process, we will obtain informed consent from the responsible authorities for their health facilities to take part in the study.

8.3.2 Recruitment of individual participants

Participants will be recruited from among women attending antenatal care (ANC) clinics at health facilities supported by FACES. Health facilities will be included based on receiving FACES support to offer PMTCT services. We plan to enroll participants who are a close representation of the real-world target population. We will recruit all HIV-positive pregnant women enrolled in the PMTCT program at the target health facilities.

8.3.3 Individual participant inclusion criteria

This study is designed as a pragmatic trial in order to estimate the effectiveness of the TextIT intervention under real world conditions [38]. Therefore, we do not have stringent participant eligibility criteria. We will enroll all participants who meet the following minimal criteria:

1. are ≥18 years or emancipated minors;
2. are at 28 weeks gestation or greater (or have delivered on the day of enrolment);
3. provide informed consent.

Women who report sharing phones will be enrolled only if they have disclosed their HIV serostatus to the person with whom the phone is shared. Of note, we were able to successfully achieve this in our formative trial (KEMRI SSC No. 2103). Women who require counseling on assisted disclosure will be referred to an on-site nurse-counselor. We will collect data on the proportion and characteristics of women who don't have access to phones or cannot participate for other reasons. These additional data will be especially important for informing the anticipated countrywide scale-up by the Kenya National AIDS/STI Control Program.

8.3.4 Exclusion criteria

We will exclude women who report sharing phones but have not disclosed their HIV status to the person with whom the phone is shared. In addition, women who do not meet any of the inclusion criteria will also be excluded.

8.4 Randomization Strategy and Sequence generation

Random allocation of the TextIT intervention periods will be done at the cluster level. Health facilities will be stratified by patient volume as measured by the average number of pregnant women newly enrolled into the PMTCT program in the 12 months prior to randomization: ≥50 new enrollments per month versus <50 new enrollments. A randomization scheme will be computer-generated for each stratum. Half of the clusters will then be randomly assigned to receive the TextIT intervention starting from the first time period, and the other half assigned to receive the intervention starting from the second time period.

8.5 Allocation concealment

The treatment assignment for clusters will be retained by the study biostatistician until PMTCT program staff at all health facilities have undergone comprehensive training on the TextIT intervention. Those who are in the control condition at time 1 will receive training when they begin implementing the intervention at time 2.

8.6 Implementation of randomization procedures

Enrolled clusters will be stratified into two groups according to the average number of women newly enrolled in PMTCT in the previous year (≥50 versus <50 new enrollments). An independent biostatistician at the University of Washington's Center for AIDS Research Biometrics Core will generate the randomization sequence for each stratum and assign clusters to the different intervention starting periods. Thereafter, the biostatistician will email the treatment allocation list to the Principal Investigator. The biostatistician will not be involved in any other aspects of the study conduct.

All women who present to the selected sites and are determined to be enrolled in the PMTCT program and pregnant at 28 weeks gestation or greater will be included in the clusters. Informed consent to collect data will be sought from individual women at the time when they present to the clinic and are found eligible for inclusion in the intervention clusters.

8.7 Blinding

Due of the nature of this intervention, it will not be possible to blind clusters, health care providers, investigators, or individual participants to the group assignments.

8.8 Sample size determination

The outcomes from primary objectives 1 and 2 are binary on the individual level, and the primary analysis for each objective will estimate a relative risk comparing the proportion who attended the postpartum clinic visit (mothers) or were HIV-tested (infants) in the intervention condition compared to the standard condition.

For a stepped-wedge design with *I* clusters, *T* time points, and *N* individuals sampled per cluster per time interval, let Y_{ijk} be the value of the outcome (0 or 1) in individual *k* at time *j* from cluster *i* (*i* in 1, …, *I; j* in 1, …, *T*; *k* in 1,…, *N*). Let X_{ij} be an indicator of the treatment assignment in cluster *i* at time *j*, with 0=control and 1=intervention. The primary measure of treatment effect is a log relative risk denoted θ; the estimate of the treatment effect is $\hat{\theta}$.

Power:

We used the methods described by Hussey and Huges [39] to determine power to test the hypothesis $H_0: \theta = 0$ versus $H_A: \theta \neq 0$ for outcomes 1 and 2. A Wald test of this hypothesis may be based on $Z = \hat{\theta} / \sqrt{\mathrm{Var}(\hat{\theta})}$. It can be shown that:

$$\mathrm{Var}(\hat{\theta}) = \frac{I\sigma^2(\sigma^2 + T\tau^2)}{(IU - W)\sigma^2 + (U^2 + ITU - TW - IV)\tau^2}$$

where $U = \sum_{ij} X_{ij}$, $W = \sum_j (\sum_i X_{ij})^2$, $V = \sum_i (\sum_j X_{ij})^2$, σ^2 is the within-cluster variance, and τ^2 is the between-cluster variance. The approximate power for conducting a two-tailed test of size α is given as:

$$\text{power} = \Phi\left(\frac{\theta_A}{\sqrt{\text{Var}(\hat{\theta})}} - Z_{1-\alpha/2}\right)$$

where Φ is the cumulative standard Normal distribution function and $Z_{1-\alpha/2}$ is the (1- α/2)th quantile of the standard Normal distribution function.

We propose a study with *I* = 20, *T* = 2, mean *N* = 57, and total sample size of 2,280. We assume a coefficient of variation (τ/μ) of 0.25 and calculate the power for a two-tailed test with α=0.05. For Outcome 1, we anticipate that 12% of women in the control group will attend a postpartum clinic visit within eight weeks after delivery [35]. Under these assumptions, we will have 90% power to detect an increase in the attendance rate in the intervention group to 20% or greater. For Outcome 2, assuming that 40% of infants in the control group will undergo virological HIV testing [35], we will have 92% power to detect an increase to 53% or greater in the intervention group. Presuming 10% loss to follow-up, we propose a sample size of 2,508 women.

Our proposed sample size of 2,508 women is based upon the need to obtain sufficient power to detect a significant difference between the two time periods. However, because the TextIT intervention will be offered to all women who are eligible at the target health facilities and is expected to be established as a routine program, we expect to continue enrolling women to receive the intervention beyond the target sample size. Therefore, we will enroll a minimum of 2,508 women and we will not have an upper limit for the sample size. At the point when we reach our minimum sample size, the FACES program will support the text messaging intervention and enroll women as part of their routine programmatic work.

8.9 STUDY PROCEDURES

Pregnant women attending ANC are tested for HIV according to the Kenya Ministry of Health guidelines. Those testing positive are provided with PMTCT services. They are counseled to visit the ANC at least three more times during their pregnancy (at 20-24 weeks, 28-32 weeks and at 36 weeks). We will enroll women either 1) ante-natally at ≥ 28 weeks gestation (as determined by date from last normal menstrual period) or 2) at the time of delivery. After obtaining verbal informed consent for participants at facilities randomized to the TextIT arm, participant baseline clinical and demographic characteristics will be recorded. Participants at facilities randomized to the TextIT arm will then be registered to receive intervention messages.

8.9.1 Participant registration

Registration will involve sending a text message in a pre-defined format that will include the participant's study identification number, date of the last normal menstrual period, preferred time of day, and language in which they would like to receive messages (from a choice of English, Kiswahili, or Dholuo). Additionally, these women will have the option of providing a preferred name to be included in outgoing messages.

8.9.2 The TextIT intervention

Registered women will then receive up to 14 text messages as follows: weeks 28, 30, 32, 34, 36, 38, 39, and 40 during the third trimester of pregnancy; weeks 1, 2, 3, 4, 5, and 6 after delivery. The message content and schedule is presented in appendix 4 below and is adapted from an earlier study that determined the efficacy of this intervention [35]. We have already developed an automated text messaging software and have further upgraded it to support large scale roll-out. Each participant's phone will be loaded with Kenya Shillings 20 (approximately 0.25 US$) to cover the cost of the registration message.

Participants at facilities receiving TextIT will have the option to call or send text messages to a designated clinic phone, to which a clinic nurse will respond. Participants will also have an option to request a call from the clinic by sending a free "call back" text message to the designated clinic phone at any time. If a participant requests to be called back, our automated software will acknowledge the request by sending back a message in the participant's preferred language containing the text, "Thank you. A clinician will call you soon." At the same time, the software will send a message to the clinic nurse on call containing the text, "Patient [name] has asked to be called at [phone number]." Upon receiving this message, the nurse will then call the participant and record details of the conversation on a standardized clinic form. An experienced clinical provider from the FACES program will be available for consultation by the study nurse at any time through the *Uliza!* consultation service. *Uliza!* is a toll-free 24-hour telephone consultation service for HIV clinical service providers in Kenya that is run by FACES in partnership with the Kenya National AIDS and STI Control Program [40].

8.9.3 Ascertainment of delivery status

At or around the estimated time of delivery (starting from the 37th week of gestation), clinic staff will check clinic records and call participants at facilities receiving the TextIT intervention to ascertain whether delivery has occurred. If delivery is confirmed to have occurred, clinic staff will record the date, outcome, place and mode of delivery, and whether intrapartum/postpartum antiretroviral prophylaxis is being received. Additionally, the baby's sex and name will be ascertained and the information used to update subsequent text messages. Participants who will not have delivered will be called again a week later and every week thereafter until delivery status is ascertained. In order to ascertain delivery status for participants at facilities receiving standard care (control group), clinic staff will check clinic records daily, beginning from the estimated date of delivery, and abstract relevant data. For all participants, if no record of delivery exists at the facility at the end of the follow-up period, clinic staff will obtain this information at the first postnatal contact with the mother (for women who visit the clinic) or by making a phone call at the end of the study follow-up period.

8.9.4 Study withdrawal

Participants will be free to withdraw from the study at any time by presenting to the clinic and indicating their desire to withdraw, or by sending an SMS with the word 'STOP'. Reasons for study withdrawal will be ascertained either at the study clinic or by telephone. Where possible, information on study endpoints will be evaluated at the time of study withdrawal. No further study related messages will be sent, or phone calls made, to those who withdraw.

8.9.5 Outcome ascertainment

The primary outcome measures will be: 1) maternal postpartum PMTCT retention (defined as documented return for at least one visit at the PMTCT, postnatal or general HIV care clinic within eight weeks after delivery); 2) virological infant HIV testing (defined as obtaining a dried blood spot sample for HIV-Polymerase Chain Reaction testing by eight weeks after birth).

In Kenya, the Ministry of Health recommends three postnatal clinic visits: one within 48 hours, another within one to two weeks, and a third at 6 weeks [41]. Maternal postpartum PMTCT retention will be assessed by abstracting information from patient charts, clinic records, and laboratory registers. A positive outcome will include the existence of a dated medical record documenting a clinic visit within eight weeks after delivery. Mothers enrolled in PMTCT programs are asked to bring their infants to clinic at 6 weeks postpartum for infant HIV testing. Virological infant HIV testing will be assessed by abstracting information from medical records including the HIV exposed infant follow-up register, HIV exposed infant card, ANC register, ANC booklet, postnatal clinic register, laboratory DBS register, patient clinic charts, and electronic medical records. At FACES, women who fail to attend clinic appointments are traced intensively to ascertain reasons for appointment default and to engage them back in care. We will rely on this well-established tracing program to ascertain birth and other outcomes for women who are lost to follow-up.

Additional outcomes to be assessed include, but are not limited to: place of delivery; skilled birth attendance (delivery at a health facility or with a skilled attendant present if not at a health facility); a combined outcome for stillbirth (after 28 weeks of pregnancy) or infant death within the first two months after a live birth; birth weight; reported infant feeding option; and incidence rate of HIV-1 among infants who undergo virological HIV testing. Follow up for mother-baby pairs in PMTCT programs typically ends at 18 months at which time the child's HIV status is confirmed by antibody testing. Possible outcomes at 18 months include: discharge from the PMTCT program (if confirmed HIV negative); referral to the general HIV comprehensive care clinic (if confirmed HIV positive); transfer out to another health facility; lost to follow-up; or death. Pending additional funding, we will also aim to assess these outcomes for mother-baby pairs at 18 months.

There will be a final "exit" phone interview: for mothers who will have not returned by 8 weeks post-partum, we will find out reasons for failure to return; for all mothers in the TextIT group, we will assess satisfaction with the text messages.

8.9.6 Limitations

Outcomes for women who attend postnatal clinic outside the study area might be difficult to determine. As part of this study, we will strengthen the already existing mechanisms for tracking clinic defaulters in order to capture outcome data for a high proportion of participants. Also, if participants change phone numbers or lose their phones, some messages may not reach them. Therefore, participants will be asked to provide alternate phone numbers if available.

8.9.7 TextIT Advisory Group

An advisory group will be formed to carry out selection of health facilities for inclusion and to promote linkages with the Ministry of Health, county level departments of health, local communities, and other stakeholder groups. This advisory group will be composed of: 1) the investigators; 2) two PMTCT experts from FACES; 3) a biostatistician; 4) representatives from the ministry of health; and 5) a community health worker.

9.0 ETHICAL CONSIDERATIONS

9.1 Informed Consent

The County Governments in the target counties of Kisumu, Homa Bay, and Migori have indicated their support for the expansion of this intervention as part of standard care. The Family AIDS Care and Education Services (FACES) has also recently completed a pilot of this intervention within the same region. Following the pilot, FACES has included this text messaging intervention as part of routine care for pregnant and post-partum HIV-positive women attending care at all health facilities that they support, including the study sites for this research. Therefore, this intervention will be delivered as part of routine standard care. In this context, the usual practice at Ministry of Health facilities is to obtain verbal consent. As such, although this intervention will be at the health facility level, we will seek individual patient verbal consent for participants at health facilities implementing the TextIT intervention. All eligible women will be provided with information about the research and the potential social risks. We will then document in our study records that they have indeed consented prior to enrolment. The information about the study will describe the study procedure in the language the subject is comfortable with (English, Swahili or Dholuo). The subject will have an opportunity to ask questions and health facility staff will be on hand to answer any questions that arise, as is the case with other interventions offered as part of routine standard care.

For participants who will be receiving usual care during the first “step” of the research, we already have continuing approval from KEMRI ERC for evaluation of program data captured routinely between September 2004 and June 2015 within FACES (NRP 1/2009). Participants at these facilities will not receive any study related intervention and so data collection activities will be limited to de-identified data that is approved for collection under the existing KEMRI approval (NRP 1/2009).

9.2 Ethical Approval

Ethical approval for this study will be sought from the Kenya Medical Research Institute’s (KEMRI) Ethical Review Committee (ERC), the University of Washington’s Human Subjects Division and the UCSF Committee on Human Research. KEMRI’s review system will involve an initial review of the protocol by the Center for Microbiology Research Scientific Committee. Upon approval, the protocol will be submitted to the KEMRI Scientific Steering Committee to be reviewed for scientific merit. If approved, the protocol will be forwarded to the KEMRI Ethics Review Committee for final ethical approval.

9.3 Protecting privacy and confidentiality

All education, counseling and testing will be done as stipulated in the Kenya national guidelines for the prevention of mother-to-child transmission of HIV. All participants will be identified using an identification code that will be the same as the client number assigned to them at the clinic. None of the clinic procedures are study procedures. Our study specifically seeks to obtain approval to access client records and to link the data to our study identification number.

All data, including the computer containing the automated SMS software and databases, will be locked in a secure room with access restricted only to authorized personnel. Similarly, all computers containing study data will be password protected and only accessible to authorized staff.

All staff members who will carry out study procedures will undergo training on research ethics and Good Clinical Practice (GCP) through the online Collaborative Institutional Training Initiative (CITI) prior to initiation of the trial. Certificates of successful completion of GCP training will be kept in the trial master file on site.

9.4 Potential risks of proposed research to study subjects

We anticipate minimal risk to study participants in this study. No biological specimen will be collected as part of the study.

There exists the potential for exposing a participant's personal information to others, especially if phones are shared. However, the study will not send out any sensitive personal health information. Further, study procedures will be thoroughly explained to participants and they will be consented only if they fully understand study procedures and find the messages acceptable to them. Participants are also allowed to exit from the study at any time. Participants who share phones will only be enrolled if they have disclosed their HIV status to the person with whom the phone is shared.

Patient anonymity may be compromised since the phone numbers to which messages are sent are essentially personal identifiers. However, the computer on which these phone numbers will be stored will be accessible only to specifically designated staff. Every effort will be made to safeguard subjects from future contact. The electronic databases will not contain participant names. Health information will be protected by coding all information required for the study and keeping the coded data separate from clinic records. The list of coded and uncoded information (linkage sheet) will also be kept separately. This linkage sheet will be maintained for a maximum of five years, after which all links between coded and uncoded data will be broken. All databases will be password protected and accessible only to authorized staff.

Because we will be accessing clinic records, there is a risk of breach of confidentiality. All staff involved in the study will undergo training in order to ensure that confidentiality of patient data is maximized.

9.5 Potential benefits of proposed research to study subjects and others

This study seeks to expand an intervention that has previously been found efficacious for improving postpartum retention in PMTCT and uptake of early infant diagnosis. Retention in

PMTCT care through the postpartum period and early determination of infant HIV status ensures timely initiation of life-saving antiretroviral therapy (for HIV positive infants) or prophylaxis (for HIV negative breastfeeding infants). Moreover, it provides an additional opportunity for important counseling and support on infant feeding options. We anticipate that these benefits will accrue to all study subjects.

Knowledge gained from this study will help inform policy makers and HIV prevention program implementers on strategies to further expand this efficacious intervention. Specifically, this study will provide an understanding of the effectiveness of the intervention (and its limitations) in a real-world, routine care setting.

10.0 DATA MANAGEMENT

10.1 Data Collection

Patient level data will be collected using standard study forms supplemented by existing clinic charts, registers provided by the Ministry of Health (e.g. antenatal care register, postnatal care register, HIV-exposed infants register, maternity register), and electronic medical records. All data will be transferred into a secure electronic study database. Data will be collected continuously. In order to minimize the costs of using paper and to ensure data integrity, we propose to use a mobile phone/tablet based data collection platform. This will be based on the Open Data Kit (ODK) Collect, a free and open source mobile phone based data collection platform that we are successfully using for other research studies at FACES. Using this system will likely also improve data integrity at the health facilities by enforcing logic and range checks at the point of data entry. Data entry will be done by designated clinic staff members who will have undergone training on how to use the study databases as well as on protection of human subjects.

10.2 Data Storage and Security

All study computers and records will be stored in a secure room with access limited only to authorized staff. All computers and databases will be password-protected with limited access.

10.2.1 Statistical Analysis

Descriptive analyses

Statistical analyses will be performed using STATA software (StataCorp, College Station, TX). The flow of clusters and participants through the study will be reported using a “CONSORT” diagram modified for cluster randomized trials [42]. At the cluster level, we will report the numbers of health facilities selected for inclusion, randomly assigned to either the intervention or control group, received the intervention, and were included in the analysis for primary outcomes. We will also report the number of health facilities excluded and the reasons for exclusion. At the participant level, we will report the number of women screened, those who meet inclusion criteria, those excluded (and reasons for exclusion), and the number within each cluster at each time period. Baseline characteristics at the cluster and individual level will be summarized using a table.

Inferential analyses

Inferential analysis for the primary outcomes will follow the intention-to-treat principle. All participants will be analyzed according to the cluster randomization group at the time of enrollment, and will be considered exposed to the intervention group regardless of whether they receive the intervention or not. Women attending clinics assigned to the TextIT group who lack phones will continue to receive standard care. However, they will be considered as having been exposed to the TextIT intervention in the intent-to-treat analysis.

Our primary analyses will be on the individual-level binary response values, $Y_{ijk,}$ for both primary outcomes. The predictor of interest will be treatment assignment (cluster-level values X_{ij}) and time period *j* will be included as a covariate. Our analyses will use generalized estimating equations (GEE) methods on the individual-level data to account for variable cluster sizes. Such methods are robust to misspecification of the variance structure when the “sandwich” estimate of the variance is used [39]. Specifically, we will use modified binomial regression with a log link and robust variance estimation to estimate the relative risk and 95% confidence intervals, using GEE with working independence correlation structure to account for clustering by site. All tests will be conducted at the 5% significance level (two-sided).

Cost-effectiveness analysis

The cost of all the components of the intervention will be measured using standard microcosting techniques [43]. All resources required to deliver the TextIT intervention will be inventoried and categorized into standard cost categories: personnel, supplies, consumables, services, capital goods, space, and overheads. Other clinical service costs that cannot be directly estimated will be from the WHO CHOICE database. We will assess costs before and after TextIT in order to compare it with the standard of care and calculate the total per-patient cost.

We will calculate the cost per disability-adjusted life year (DALY) averted comparing current standard of care and the TextIT intervention using the incremental cost effectiveness ratio (ICER). The ICER is defined as the cost difference between current standard of care and TextIT divided by difference in DALYs. The health effects (DALYs) averted due to the intervention among infants will be assessed using the methods described in the Global Burden of Disease Study (GBD) [44, 45], by adding years of healthy life lost due to premature mortality (YLL) and years lived with disability (YLD). The YLL will be estimated as a product of the number of deaths and the life expectancy at age of death. The YLD will be estimated as a product of the prevalence of HIV among infants and the disability weights from the GBD. Key inputs for effectiveness of the intervention will be determined from the inferential analyses described above. This analysis will take the perspective of a policy maker (NASCOP).

Analyses will be done using Stata software (StataCorp, College Station, TX). No interim analyses are planned. A detailed formal statistical analysis plan will be developed prior to data analysis.

Analysis of implementation outcomes and public health impact

Because implementation outcomes are interrelated and have complex interactions [1], we propose to use the RE-AIM framework whose constructs summarize what we feel are important

dimensions of an intervention aimed at rapidly translating research into practice in a high HIV prevalence, resource limited setting. Using the RE-AIM framework [32, 46], we will calculate the reach, effectiveness, adoption, implementation, and maintenance dimensions of the TextIT intervention. This approach has been used to evaluate the dissemination of various public health interventions [31, 47].

Reach refers to the level of penetration of an intervention in terms the proportion of eligible participants who receive the intervention [1]. In our case, the denominator for calculating Reach – the total eligible population – will be the total number of HIV-positive pregnant women in their third trimester of pregnancy attending care or who deliver at the study health facilities during the intervention period (all of whom will be contacted for study participation). Women who decline study participation will still be included in the denominator. This information will be abstracted from the ANC registers, PMTCT registers, maternity registers, and electronic medical records. The numerator – the number of persons who receive the intervention – will be the total number of women who are registered to receive the TextIT intervention.

Effectiveness – an intervention's impact on targeted outcomes – will be the effect size of the intervention on the primary outcomes comparing the two study arms. To gain additional insight into effectiveness, we will conduct qualitative "exit" interviews with women who receive the TextIT intervention to assess satisfaction with the intervention.

Adoption – the proportion of organizational units or settings that adopt a given intervention – will be measured as the proportion of health facilities in which the TextIT strategy will have been implemented by the end of the study out of all the health facilities initially approached to take part in the study. In order to better understand the construct of adoption, we will further compare the characteristics of participating versus non-participating health facilities and present descriptive statistics (proportions and frequencies).

Implementation – the delivery of an intervention with fidelity to the original design – will be measured at the participant level as the proportion of women registered to receive messages to whom messages are sent as scheduled. This will be determined from automated reports of outgoing text messages generated by our TextIT SMS software.

Maintenance – the sustainability or institutionalization of an intervention – is difficult to measure in that it requires long-term follow-up. In this study, we will measure a proximal indicator of maintenance at the organizational level as the proportion of health facilities that include the TextIT strategy in their annual operating plan – a resource planning document that each health facility is required to prepare every year – for the financial year following the formal conclusion of the study.

In order to present a summary metric of the public health impact, we will combine the RE-AIM dimensions as proposed by Glasgow et al [46]. To determine individual level impact, we will compute the product of the Reach and Effectiveness dimensions (Reach x Effectiveness) to yield an RE index. This composite index provides a summary measure of the extent of penetration of the intervention in relation to the effect size. To determine the population level impact, we will compute the product of Adoption and Implementation (Adoption x

Implementation). This index provides a summary of the fidelity with which the program is implemented as intended combined with the proportion of health facilities that adopt the TextIT intervention.

In order to achieve fidelity to the RE-AIM implementation model, we plan to report our evaluation according to the criteria for "fully developed use" of RE-AIM as proposed by Kessler et al [48]. We will aim to report all the core items that are envisioned in each of the five RE-AIM dimensions.

11.0 TIME FRAME

This project will take approximately 24 months to complete. Because the proposed study will take place at established sites that already have years of experience with HIV service provision under CDC/PEPFAR funding, it is expected that startup activities will be fully integrated into the health facilities within the first six months, including training of health facility staff. We will allow six months to set up the project, twelve months to implement the intervention and collect data, three months to analyze the data and prepare the primary report of our findings, and a buffer period of three months to address any unanticipated logistical challenges, which we almost invariably encounter in this type of international research.

	JAN – DEC 2014			JAN – DEC 2015	
	Month 1–2	Months 3–6	Months 7–12	Months 13–18	Months 19–24
IRB applications (Kenya and US)	●	●			
Hire & train staff; study launch	●	●			
Community engagement meetings		●	●		
Study enrollment			●	●	
Participant follow-up			●	●	●
Data analysis					●
Dissemination of findings					●

12.0 EXPECTED APPLICATION OF THE RESULTS

This study will provide an understanding of the effectiveness of a novel strategy to improve adherence to postnatal clinic visits and increase the uptake of infant HIV testing in a real-world, routine-care setting. Findings from this study will guide the development of protocols, training manuals, implementation procedures, data collection forms, and electronic databases that would allow for nationwide expansion of the intervention. This would also allow other PMTCT programs to implement the intervention independently and with high fidelity to our model.

We will share the findings from this study with the health facilities involved, community leaders, County Ministers of Health, and NASCOP. Moreover, we will disseminate our findings at local, national, regional, and international meetings and conferences.

13.0 REFERENCES

1. Proctor E, Silmere H, Raghavan R, Hovmand P, Aarons G, Bunger A, Griffey R, Hensley M: **Outcomes for implementation research: conceptual distinctions, measurement challenges, and research agenda.** *Adm Policy Ment Health* 2011, **38:**65-76.
2. **United Nations General Assembly Sixty-fifth Session Agenda Item 10. Political Declaration on HIV/AIDS: Intensifying our Efforts to Eliminate HIV/AIDS** [http://www.unaidsrstesa.org/prvs/sites/default/files/political_declaration_english.pdf]
3. **Global report: UNAIDS report on the global AIDS epidemic 2012** [http://www.unaids.org/en/media/unaids/contentassets/documents/epidemiology/2012/gr2012/20121120_UNAIDS_Global_Report_2012_with_annexes_en.pdf]
4. Rawizza H, Meloni S, Oyebode T, Sagay S, Adewole I, Okonkwo P, Kanki P, Team AP: **Evaluation of Loss to Follow-up within the PMTCT Care Cascade in a Large ART Program: Nigeria.** In *Book Evaluation of Loss to Follow-up within the PMTCT Care Cascade in a Large ART Program: Nigeria* (Editor ed.^eds.). City; 2012.
5. Nassali M, Nakanjako D, Kyabayinze D, Beyeza J, Okoth A, Mutyaba T: **Access to HIV/AIDS care for mothers and children in sub-Saharan Africa: adherence to the postnatal PMTCT program.** *AIDS Care* 2009, **21:**1124-1131.
6. Perez F, Orne-Gliemann J, Mukotekwa T, Miller A, Glenshaw M, Mahomva A, Dabis F: **Prevention of mother to child transmission of HIV: evaluation of a pilot programme in a district hospital in rural Zimbabwe.** *BMJ* 2004, **329:**1147-1150.
7. Mirkuzie AH, Hinderaker SG, Morkve O: **Promising outcomes of a national programme for the prevention of Mother-to-Child HIV transmission in Addis Ababa: a retrospective study.** *BMC Health Serv Res* 2010, **10:**267.
8. **Policy requirements for HIV testing and counselling of infants and young children in health facilities.** In *Book Policy requirements for HIV testing and counselling of infants and young children in health facilities* (Editor ed.^eds.). City: World Health Organization and UNICEF; 2010.
9. **Global HIV/AIDS Response: Epidemic update and health sector progress towards Universal Access** [http://www.unaids.org/en/media/unaids/contentassets/documents/unaidspublication/2011/20111130_UA_Report_en.pdf]
10. Cook RE, Ciampa PJ, Sidat M, Blevins M, Burlison J, Davidson MA, Arroz JA, Vergara AE, Vermund SH, Moon TD: **Predictors of successful early infant diagnosis of HIV in a rural district hospital in Zambezia, Mozambique.** *J Acquir Immune Defic Syndr* 2011, **56:**e104-109.
11. Braun M, Kabue MM, McCollum ED, Ahmed S, Kim M, Aertker L, Chirwa M, Eliya M, Mofolo I, Hoffman I, et al: **Inadequate coordination of maternal and infant HIV services detrimentally affects early infant diagnosis outcomes in Lilongwe, Malawi.** *J Acquir Immune Defic Syndr* 2011, **56:**e122-128.
12. Dube Q, Dow A, Chirambo C, Lebov J, Tenthani L, Moore M, Heyderman RS, Van Rie A: **Implementing early infant diagnosis of HIV infection at the primary care level: experiences and challenges in Malawi.** *Bull World Health Organ* 2012, **90:**699-704.
13. Becquet R, Marston M, Dabis F, Moulton LH, Gray G, Coovadia HM, Essex M, Ekouevi DK, Jackson D, Coutsoudis A, et al: **Children Who Acquire HIV Infection Perinatally Are at Higher Risk of Early Death than Those Acquiring Infection through Breastmilk: A Meta-Analysis.** *PLoS One* 2012, **7**.
14. Mutel T, Akonde A, Doumbia A, Traore DC, Diarra M, Maiga F, Coulibaly B, Pizarro L, Perez F: **The prevention of mother-to-child transmission of HIV in Mali: HIV-positive pregnant women and loss to follow-up in the Segou region. Abstract MOPE480.** In *Book The prevention of mother-to-*

child transmission of HIV in Mali: HIV-positive pregnant women and loss to follow-up in the Segou region. Abstract MOPE480 (Editor ed.^eds.). City; 2011.
15. Peltzer K, Mlambo G: **Factors determining HIV viral testing of infants in the context of mother-to-child transmission.** *Acta Paediatr* 2010, **99:**590-596.
16. Chang LW, Kagaayi J, Arem H, Nakigozi G, Ssempijja V, Serwadda D, Quinn TC, Gray RH, Bollinger RC, Reynolds SJ: **Impact of a mHealth intervention for peer health workers on AIDS care in rural Uganda: a mixed methods evaluation of a cluster-randomized trial.** *AIDS Behav* 2011, **15:**1776-1784.
17. Mahmud N, Rodriguez J, Nesbit J: **A text message-based intervention to bridge the healthcare communication gap in the rural developing world.** *Technol Health Care* 2010, **18:**137-144.
18. Seidenberg P, Nicholson S, Schaefer M, Semrau K, Bweupe M, Masese N, Bonawitz R, Chitembo L, Goggin C, Thea DM: **Early infant diagnosis of HIV infection in Zambia through mobile phone texting of blood test results.** *Bull World Health Organ* 2012, **90:**348-356.
19. **Opening remarks by UNICEF Regional Director Elhadj As Sy at the Global Fund PMTCT meeting** [http://www.unicef.org/esaro/5440_6380.html]
20. Besser M, Nolan M, Fulton N, Goheen M, Simba M: **Promoting client retention during PMTCT and follow-up care: lessons learned through active client follow up at mothers2mothers sites in Kenya and South Africa.** In *Book Promoting client retention during PMTCT and follow-up care: lessons learned through active client follow up at mothers2mothers sites in Kenya and South Africa* (Editor ed.^eds.). City; 2010.
21. Besser M, Sogaula N, Goheen M, Myers A, Nolan M: **Improving uptake of early infant HIV diagnosis through simple interventions: lessons learned at mother2mother's innovation center, South Africa.** In *Book Improving uptake of early infant HIV diagnosis through simple interventions: lessons learned at mother2mother's innovation center, South Africa* (Editor ed.^eds.). City; 2010.
22. Technau K-G, Tolly KD, Sherman G, Kuhn L, Benjamin P, Bassett J, Coovadia A: **Mobile text messaging improves PMTCT follow-up in South African public setting.** In *Book Mobile text messaging improves PMTCT follow-up in South African public setting* (Editor ed.^eds.). City; 2011.
23. **Kenya Strategic Framework for Elimination of Mother-to-Child Transmission of HIV and Keeping Mothers Alive 2012-2015.** In *Book Kenya Strategic Framework for Elimination of Mother-to-Child Transmission of HIV and Keeping Mothers Alive 2012-2015* (Editor ed.^eds.). City: Kenya Ministry of Health; 2012.
24. National AIDS and STI Control Programme MoH, Kenya: ***Kenya AIDS Indicator Survey 2007: Final Report*** In *Book Kenya AIDS Indicator Survey 2007: Final Report* (Editor ed.^eds.). City.
25. Turan JM, Bukusi EA, Onono M, Holzemer WL, Miller S, Cohen CR: **HIV/AIDS stigma and refusal of HIV testing among pregnant women in rural Kenya: results from the MAMAS Study.** *AIDS Behav* 2011, **15:**1111-1120.
26. Tomlinson M, Rotheram-Borus MJ, Swartz L, Tsai AC: **Scaling Up mHealth: Where Is the Evidence?** *PLoS Med* 2013, **10:**e1001382.
27. Free C, Phillips G, Watson L, Galli L, Felix L, Edwards P, Patel V, Haines A: **The Effectiveness of Mobile-Health Technologies to Improve Health Care Service Delivery Processes: A Systematic Review and Meta-Analysis.** *PLoS Med* 2013, **10:**e1001363.
28. The PME: **A Reality Checkpoint for Mobile Health: Three Challenges to Overcome.** *PLoS Med* 2013, **10:**e1001395.
29. Flay BR, Biglan A, Boruch RF, Castro FG, Gottfredson D, Kellam S, Moscicki EK, Schinke S, Valentine JC, Ji P: **Standards of evidence: criteria for efficacy, effectiveness and dissemination.** *Prev Sci* 2005, **6:**151-175.

30. Glasgow RE: **eHealth evaluation and dissemination research.** *Am J Prev Med* 2007, **32:**S119-126.
31. Glasgow RE, Eckstein ET, Elzarrad MK: **Implementation science perspectives and opportunities for HIV/AIDS research: integrating science, practice, and policy.** *J Acquir Immune Defic Syndr* 2013, **63 Suppl 1:**S26-31.
32. Glasgow RE, Vogt TM, Boles SM: **Evaluating the public health impact of health promotion interventions: the RE-AIM framework.** *Am J Public Health* 1999, **89:**1322-1327.
33. Hughes J, Goldenberg RL, Wilfert CM, Valentine M, Mwinga KG, Guay LA, Mmiro F, Stringer JS: **Design of the HIV Prevention Trials Network (HPTN) Protocol 054: A cluster randomized crossover trial to evaluate combined access to nevirapine in developing countries.** *UW Biostatistics Working Paper Series Working Paper 195* 2003.
34. Squire SB, Ramsay AR, van den Hof S, Millington KA, Langley I, Bello G, Kritski A, Detjen A, Thomson R, Cobelens F, Mann GH: **Making innovations accessible to the poor through implementation research.** *Int J Tuberc Lung Dis* 2011, **15:**862-870.
35. Odeny T, Bukusi E, Cohen C, Camlin C, Yuhas K, McClelland R: **Texting improves testing: a randomized controlled trial of text messaging to increase postpartum clinic attendance and rates of early infant diagnosis of HIV.** In *Book Texting improves testing: a randomized controlled trial of text messaging to increase postpartum clinic attendance and rates of early infant diagnosis of HIV* (Editor ed.^eds.). City: Abstract #MOPE190; 2013.
36. Torgerson DJ: **Contamination in trials: is cluster randomisation the answer?** *BMJ* 2001, **322:**355-357.
37. National AIDS and STI Control Programme MoH, Kenya: **Kenya AIDS Indicator Survey 2012: Preliminary Report** In *Book Kenya AIDS Indicator Survey 2012: Preliminary Report* (Editor ed.^eds.). City.
38. Treweek S, Zwarenstein M: **Making trials matter: pragmatic and explanatory trials and the problem of applicability.** *Trials* 2009, **10:**37.
39. Hussey MA, Hughes JP: **Design and analysis of stepped wedge cluster randomized trials.** *Contemp Clin Trials* 2007, **28:**182-191.
40. Karari C, Tittle R, Penner J, Kulzer J, Bukusi EA, Marima R, Cohen CR: **Evaluating the uptake, acceptability, and effectiveness of Uliza! clinicians' HIV hotline: a telephone consultation service in Kenya.** *Telemed J E Health* 2011, **17:**420-426.
41. Warren C, Mwangi A, Oweya E, Kamunya R, Koskei N: **Safeguarding maternal and newborn health: improving the quality of postnatal care in Kenya.** *Int J Qual Health Care* 2010, **22:**24-30.
42. Campbell MK, Piaggio G, Elbourne DR, Altman DG: **Consort 2010 statement: extension to cluster randomised trials.** *BMJ* 2012, **345:**e5661.
43. Muennig P: *Cost-effectiveness analysis in health: a practical approach.* Jossey-Bass; 2007.
44. Murray CJ, Vos T, Lozano R, Naghavi M, Flaxman AD, Michaud C, Ezzati M, Shibuya K, Salomon JA, Abdalla S, et al: **Disability-adjusted life years (DALYs) for 291 diseases and injuries in 21 regions, 1990-2010: a systematic analysis for the Global Burden of Disease Study 2010.** *Lancet* 2012, **380:**2197-2223.
45. Ortblad KF, Lozano R, Murray CJ: **The burden of HIV: insights from the GBD 2010.** *AIDS* 2013.
46. Glasgow RE, Klesges LM, Dzewaltowski DA, Estabrooks PA, Vogt TM: **Evaluating the impact of health promotion programs: using the RE-AIM framework to form summary measures for decision making involving complex issues.** *Health Educ Res* 2006, **21:**688-694.
47. Brownson RC, Colditz GA, Proctor EK: *Dissemination and implementation research in health: translating science to practice.* Oxford University Press; 2012.
48. Kessler RS, Purcell EP, Glasgow RE, Klesges LM, Benkeser RM, Peek CJ: **What does it mean to "employ" the RE-AIM model?** *Eval Health Prof* 2013, **36:**44-66.

14.0 APPENDICES

14.1 Participant Information Statement – English

14.2 Participant Information Statement – Kiswahili

14.3 Participant Information Statement – Dholuo

14.4 Baseline questionnaire

Baseline Questionnaire

Informed Consent:
Please note: informed consent must be given before any study specific procedures take place.

Has the participant provided verbal informed consent? Yes ☐ No ☐

SMS Preferences:

1. Mobile phone number(s): (a) ☐☐☐☐☐☐☐☐☐☐ (b) ☐☐☐☐☐☐☐☐☐☐
2. Best time to send SMS: ☐☐☐☐ hours
3. Preferred SMS language: ☐ English ☐ Dholuo ☐ Kiswahili
4. Is mobile phone shared? ☐ Yes ☐ No

Demographics:

5. (a) Age (yrs): ☐☐ (b) Date of Birth: ☐☐ / ☐☐☐ / ☐☐
6. What is the location of your residence? (village, estate or sub-location) ________________________________
7. What is your ethnic origin? (check one box)
   ☐ Luo ☐ Other (please specify): ________________________________
8. Are you currently employed? (check one box)
   ☐ Yes ☐ No
9. What is the highest level of school education you completed? (check one box)
   ☐ No level completed
   ☐ Primary
   ☐ Secondary
   ☐ Post Secondary

10. What is your current marital status? (check one box)
    ☐ Not married, without regular live-in partner
    ☐ Not married, with a regular live-in partner
    ☐ Married, not living with husband
    ☐ Married, living with husband
    ☐ Other (specify):______________________________

11. What is your religion? (check one box)
    ☐ None / I Don't Know ☐ Christian ☐ Muslim ☐ Hindu ☐ Other (please specify):__________________________

Obstetric History (from ANC register and mother-child booklet):

12. Gravidity (total number of pregnancies regardless of outcome, including current one):
    ☐☐

13. Parity (number of previous deliveries that occurred at a gestation beyond 24 weeks/6 months):
    ☐☐

14. LMP (first day of last normal menstrual period):
    ☐☐ / ☐☐☐ / ☐☐

15. EDD (estimated date of delivery)
    ☐☐ / ☐☐☐ / ☐☐

16. Gestation (duration of the pregnancy in weeks)
    ☐☐ weeks

17. Weight (measured at today's clinic)
    ☐☐ kg

18. Blood pressure (measured at today's clinic)
    ☐☐ / ☐☐ mmHg

19. Hb (Haemoglobin level at today's clinic)
    ☐☐ mg/dL

20. RPR
    ☐ Positive ☐ Negative ☐ Not done

21. WHO stage
    ☐1 ☐ 2 ☐ 3 ☐ 4

22. CD4 cell count at baseline
    ☐☐☐☐ cells/μL

23. HAART (Is participant receiving HAART for own health?)
    ☐ Yes ☐ No
    If yes, indicate date of HAART commencement ☐☐ / ☐☐☐ / ☐☐

24. AZT prophylaxis for mother issued?
    ☐ Yes ☐ No

25. AZT + 3TC + NVP for mother issued? (Delivery pack)
    ☐ Yes ☐ No

26. AZT + 3TC for mother issued? (Post-delivery pack)
    ☐ Yes ☐ No

27. Nevirapine prophylaxis for baby issued or administered?
    ☐ Yes ☐ No

28. Results of TB screening:
    ☐ No TB signs ☐ TB suspect ☐ Already on TB treatment

29. Screening for cervical cancer:
    ☐ Yes ☐ No

30. Other medical conditions in pregnancy:
    ☐ None ☐ Hypertension ☐ Diabetes ☐ Epilepsy ☐ Malaria ☐ STI/RTI
    ☐ Other (specify) ______________________

31. Intermittent presumptive therapy for malaria given?
    ☐ Yes ☐ No

32. HIV test day:
    ☐ Today ☐ Before today

33. HIV test counseling done with partner (couple counseling)?
    ☐ Yes ☐ No

Completed by:

Staff Initials: ☐☐☐ **Date:** __ __ /__ __ __ / __ __

14.5 Postnatal visit questionnaire

Postnatal Visit Questionnaire

Postnatal visit attendance:

1. Did the participant attend postnatal clinic?
   ☐ Yes ☐ No

2. Visit Date: ☐☐ / ☐☐☐ / ☐☐

Infant and delivery details (from PNC register, mother-child booklet, HEI register):

3. Date of delivery: ☐☐ / ☐☐☐ / ☐☐

4. Mode of delivery?
   ☐ Vaginal ☐ Caesarean section

5. Place of delivery?
   ☐ Home ☐ Health facility

6. What was the outcome of delivery?
   ☐ Live birth ☐ Stillbirth

7. Sex (check one box)
   ☐ Male ☐ Female

8. HEI enrollment date: ☐☐ / ☐☐☐ / ☐☐

9. Birth Weight
   ☐☐ ☐☐ grams

10. Source of referral into HEI follow-up program
    ☐Paediatric ward ☐ MCH/PMTCT ☐ CCC ☐ Maternity

11. ARV prophylaxis issued?
    ☐ Yes ☐ No

12. ARV drugs:
    ☐ sd NVP ☐ NVP + AZT + 3TC ☐ NVP for 6 weeks ☐ Extended NVP ☐ None

13. Infant adherence to ARV prophylaxis (mother's report):
    ☐ Fully adherent ☐ Missed some doses ☐ Missed all doses

14. Infant feeding options:
    ☐ Exclusive b/f ☐ Exclusive replacement feeding ☐ Mixed feeding

HIV DNA PCR testing:

15. Was DBS done (for HIV DNA PCR testing)?
    ☐ Yes ☐ No

16. Date when DBS was done: ☐☐ / ☐☐☐ / ☐☐

17. Age when DBS was done (in weeks)
    ☐☐ weeks

18. Result of HIV DNA PCR test?
    ☐ Positive ☐ Negative

19. Other outcome at exit from HEI follow-up program?
    ☐ Transferred for HAART ☐ Transferred out ☐ Dead ☐ Lost to follow-up

Completed by:

Staff Initials: ☐☐☐ **Date: __ __ /__ __ __ / __ __**

14.6 Table of intervention text messages

Gestation Week	Message
28	Hi [name]! Congratulations for visiting clinic this week! Please call or flash 0788100133 if you have questions about your pregnancy. We're here to help you!
30	Hi [name]. We wish you a good and healthy pregnancy. We are here to support you during this journey. If you have questions please call or flash 0788100133
32	Hi [name]! We would like to wish you a good day. Please remember that if you have questions about your pregnancy, you can call or flash 0788100133
34	Good day [name]! Have you visited the mother and child care clinic lately? If not, please feel welcome to visit. Call or flash 0788100133 for questions
36	Greetings [name]! We are here for you if you have any questions about your pregnancy. Please call or flash or send a please call me to 0788100133
38	Hello [name]! Have you planned where you will deliver your baby? Please call or flash 0788100133 if you have questions or want to discuss your options
39	Hi [name], We wish you a healthy pregnancy and safe delivery! If you would like to plan your delivery, call or flash or send please call me to 0788100133
40	Hi [name], We wish you a healthy pregnancy and safe delivery! If you would like to plan your delivery, call or flash or send please call me to 0788100133

Weeks after delivery	Message
1	Dear [name], congratulations on the birth of baby [babyname]! We treasure you both! If you have questions about baby’s health please call or flash 0788100133
2	Dear [name], your baby [boy/girl] will need immunization at 6 weeks of age to prevent childhood diseases and grow healthy and strong. Please call or flash 0788100133 to find out more about baby’s clinic schedule. Thank you
3	Hi [name]! How are you & baby [babyname]? We know you're working hard to care for [babyname]. If you have any fever, pain or bleeding, please come to clinic
4	Dear [name], your baby [boy/girl] is now one month old. Congratulations! Please remember to have enough rest and sleep to keep yourself healthy
5	Hi [name]! How are you and baby [babyname]? Your baby needs to be immunized to prevent childhood diseases. Kindly bring baby to clinic next week. See you then!
6	Hi [name]! This week, please bring baby [babyname] to clinic for important immunizations to prevent childhood diseases and make sure [babyname] grows up healthy and strong. You will also be counelled on how to keep your baby [boy/girl] healthy
